# Fluorescence lifetime-based assay reports structural changes in cardiac muscle mediated by effectors of contractile regulation

**DOI:** 10.1085/jgp.202113054

**Published:** 2023-01-12

**Authors:** Alexey V. Dvornikov, Thomas A. Bunch, Victoria C. Lepak, Brett A. Colson

**Affiliations:** 1 Department of Cellular and Molecular Medicine, University of Arizona, Tucson, AZ, USA

## Abstract

Cardiac muscle contraction is regulated by Ca^2+^-induced structural changes of the thin filaments to permit myosin cross-bridge cycling driven by ATP hydrolysis in the sarcomere. In congestive heart failure, contraction is weakened, and thus targeting the contractile proteins of the sarcomere is a promising approach to therapy. However, development of novel therapeutic interventions has been challenging due to a lack of precise discovery tools. We have developed a fluorescence lifetime-based assay using an existing site-directed probe, N,N′-dimethyl-N-(iodoacetyl)-N′-(7-nitrobenz-2-oxa-1,3-diazol-4-yl)ethylenediamine (IANBD) attached to human cardiac troponin C (cTnC) mutant cTnC^T53C^, exchanged into porcine cardiac myofibrils. We hypothesized that IANBD-cTnC^T53C^ fluorescence lifetime measurements provide insight into the activation state of the thin filament. The sensitivity and precision of detecting structural changes in cTnC due to physiological and therapeutic modulators of thick and thin filament functions were determined. The effects of Ca^2+^ binding to cTnC and myosin binding to the thin filament were readily detected by this assay in mock high-throughput screen tests using a fluorescence lifetime plate reader. We then evaluated known effectors of altered cTnC-Ca^2+^ binding, W7 and pimobendan, and myosin-binding drugs, mavacamten and omecamtiv mecarbil, used to treat cardiac diseases. Screening assays were determined to be of high quality as indicated by the *Z′* factor. We conclude that cTnC lifetime-based probes allow for precise evaluation of the thin filament activation in functioning myofibrils that can be used in future high-throughput screens of small-molecule modulators of function of the thin and thick filaments.

## Teaser

Dvornikov et al. test a new high-throughput fluorescence lifetime-based assay using a human cardiac troponin C probe in cardiac myofibrils. The assay sensitivity and precision allow for the detection of key structural changes in cTnC that reports changes in thick and filament proteins in health and disease, which promises to have utility in screening therapeutic compounds that target these proteins.

## Introduction

Cardiac muscle contraction is initiated by increased cytosolic Ca^2+^ causing a conformation change in cardiac troponin C (cTnC). As a result, tropomyosin (Tm) moves from the “blocked” to “closed” position, according to the three-state model of thin filament activation (Geeves and Holmes, 1999). This allows myosin of the thick filament to bind to actin of the thin filament, further moving Tm to the “open” state. Hydrolysis of ATP by its ATPase domain causes myosin’s lever arm rotation in the actin-myosin cross-bridge power stroke to generate force and movement during contraction (Gordon et al., 2000). Dysregulation in the thin and thick filaments leads to various pathological outcomes, such as contractile dysfunction in disease.

Changes due to troponin cTnI and cTnT interactions or myosin binding to the thin filament are also propagated back to the cTnC structure, altering its conformation and affinity for Ca^2+^. Thus, Ca^2+^ binding to cTnC increases the number of force-generating actin-myosin cross-bridges and further increases Ca^2+^ binding to cTnC through cooperative feedback mechanisms (Bremel and Weber, 1972; Güth and Potter, 1987; Zot and Potter, 1989; Hannon et al., 1992). Therefore, cTnC is not a simple on-off switch regulated solely by Ca^2+^. It is found in multiple conformations that reflect thin filament activation states (Zot and Potter, 1989). These cTnC conformations can be detected by placing a fluorescent probe in the amino terminus of cTnC, in close proximity to the Ca^2+^-binding sites. Site-directed probes attached to TnC have been used to report structure–function relationships, Ca^2+^-binding kinetics, and myosin binding in thin filaments and myofibrils by monitoring fluorescence intensity (Hannon et al., 1992; Gordon et al., 2000; Davis et al., 2007; Little et al., 2011; Pinto et al., 2011; Little et al., 2012).

Myosin-binding drugs have recently been developed to treat cardiac diseases by targeting myofilament function (Malik et al., 2011; Anderson et al., 2018). Additional new drugs working at the level of the myofilaments regulatory proteins are needed, but there is currently a scarcity of high-throughput screening (HTS) platforms based on these regulatory components and their interactions in situ. Fluorescently labeled cTnC in cardiac muscle would be useful to screen for drugs that modulate protein structures in myofibrils to elicit therapeutic contractile changes for treating dysfunction in cardiac disease. However, the intensity measurements in myofibrils exchanged with fluorescent cTnC are concentration-dependent and lack the signal precision necessary for HTS. To achieve the needed separation between the fluorescence readout of different states of labeled cTnC incorporated into myofibrils for HTS, we have used an existing fluorescent probe, N,N′-dimethyl-N-(iodoacetyl)-N′-(7-nitrobenz-2-oxa-1,3-diazol-4-yl)ethylenediamine (IANBD), attached to a cysteine introduced at position 53 (cTnC^T53C^; Little et al., 2012), and measured lifetime instead of intensity. Time-resolved fluorescence (TR-F, lifetime) is proportional to intensity and monitors how many nanoseconds it takes for a population of excited fluorophores to decay from peak intensity to ∼37% (or 1/e). Compared to intensity, lifetime is largely insensitive to changes in probe concentration and improves signal precision ∼20-fold. Using labeled cTnC in myofibrils combined with a fluorescence lifetime plate reader (FLTPR) capable of precisely measuring lifetimes of thousands of samples in minutes, we have developed a novel HTS approach capable of identifying compounds impacting both the Ca^2+^-based thin filament and myosin-based thick filament regulation.

In the present study, we hypothesized that changes in human IANBD-cTnC^T53C^ lifetime due to Ca^2+^ binding to cTnC or myosin binding to the thin filament in the complex in situ environment of porcine myofibrils are indicative of the thin filament activation state. These lifetime changes are large enough and have small enough variability to be useful in HTS for compounds targeting components of the actomyosin regulatory system. These targets may include cTnC, cTnI, cTnT, Tm, actin, and myosin, among others. States of myosin tested were rigor, ADP-bound, and ATP-bound. We also monitored the effects of the Ca^2+^-troponin desensitizer W7, Ca^2+^-troponin sensitizer pimobendan (Pimo), myosin inhibitor mavacamten (Mava), and myosin activator omecamtiv mecarbil (OM) to illustrate the usefulness of this new TR-F assay in functioning myocardium.

## Materials and methods

All reagents were purchased from Fisher Scientific unless noted otherwise.

### Protein overexpression and purification of cardiac troponins

Plasmids used to express wild-type human cardiac troponin protein subunits cTnC, cTnI, and cTnT (TnT3) were provided by Dr. Jil Tardiff (University of Arizona). cTnC^T53C^ was previously developed for use in fluorescence measurements of myofilaments (Davis et al., 2007) and myofibrils (Little et al., 2012). Recombinant proteins of wild-type cTnI, wild-type cTnC, and cTnC^T53C^ were expressed in *Escherichia coli* BL21(DE3)-competent cells. Wild-type cTnT was expressed in Rosetta (DE3)-competent cells (New England Biolabs). Purification of cTnC was performed as described in Abdullah et al. (2019) using anionic exchange chromatography followed by hydrophobic interaction chromatography (HiTrap Q XL and Phenyl HP columns; GE Healthcare) and resulted in ∼95–99% purity as determined by SDS-PAGE. These cTnC fractions were concentrated in spin concentrators (Amicon Ultra, Sigma-Aldrich), dialyzed against 0.4 M KCl, and stored frozen at −80°C until use. cTnT and cTnI expression, purification, and buffers were carried out as described in Abdullah et al. (2019), except that an AKTA FPLC purification system and compatible columns were used (GE Healthcare). Briefly, cTnT was purified by cationic exchange chromatography (HiTrap Q XL column; GE Healthcare). For cTnI, following anionic exchange (HiTrap SP column; GE Healthcare), a cTnC-affinity column was used. CNBr-activated Sepharose 4B (Sigma-Aldrich) was coupled with wild-type cTnC and packed into a XK-16/20 medium pressure column (GE Healthcare).

### Labeling of cTnC^T53C^

50 μM of cTnC^T53C^ was labeled with IANBD (Thermo Fisher Scientific) in Tris-Urea-KCl-EDTA (TUKE) buffer (50 mM Tris, 6 M urea, 90 mM KCl, and 1 mM EDTA, pH 7.5). Prior to labeling, 200 μM of Tris(2-carboxyethyl)phosphine hydrochloride (TCEP; Thermo Fisher Scientific) was added and incubated for 30 min at room temperature (23°C) to reduce cysteines. Then, IANBD was added to a final concentration of 150 μM (from a 20 mM stock in dimethylformamide). Labeling was performed for ∼1 h at room temperature. The reaction was terminated by the addition of 750 μM DL-dithiothreitol (DTT; Thermo Fisher Scientific). Excess dye was removed by exhaustive dialyses against TUKE buffer and IANBD-labeled cTnC was clarified at 14,000 × *g* for 15 min at 4°C. 74 ± 3% of the cTnC cysteines were labeled with IANBD under these conditions as determined on a UV-Vis spectrophotometer.

### Preparation of cardiac troponin complex

IANBD-cTnC^T53C^ was combined with wild-type cTnI and cTnT to form the recombinant troponin complex (Tn) as described in Abdullah et al. (2019). Briefly, IANBD-cTnC, cTnT, and cTnI were individually dialyzed against Tn reconstitution buffer 1 (30 mM MOPS, 6 M urea, 0.5 M KCl, and 5 mM MgCl_2_, pH 7.0 with KOH; Sigma-Aldrich), and then mixed at a molar ratio of 1:1.2:1.2, respectively. Refolding of the Tn subunits and complex formation was carried out by dialysis against buffers with decreasing urea concentration (6 to 4 to 0 M urea). Next, Tn was dialyzed against Tn reconstition buffer 2 with KCl reduced from 0.5 to 0.4 M (30 mM MOPS, 0.4 M KCl, and 5 mM MgCl_2_, pH 7.0 with KOH). Finally, the Tn complex was dialyzed against hypotonic rigor buffer (73 mM potassium propionate, 10 mM MOPS, 10 mM EGTA, and 6 mM MgCl_2_, pH 7) used for the Tn exchange in cardiac myofibrils. Tn was then centrifuged at 14,000 rpm (18,400 × *g*) in an Eppendorf centrifuge (5424R) for 20 min at 4°C to remove uncomplexed cTnI and cTnT.

### Preparation of permeabilized myofibrils

Frozen cryoground pig ventricle heart tissue was obtained from Pel-Freez Biologicals and stored at −80°C until use. The day before the experiment, tissue was thawed in ice-cold relaxing buffer (68 mM KOH, 48 mM potassium propionate, 100 mM BES [Sigma-Aldrich], 10 mM EGTA, 6.5 mM MgCl_2_, 6.2 mM ATP [Cat. No. BP413-25], and 10 mM Na_2_CrP [Sigma-Aldrich], pH 7) containing Sigma Protease Inhibitor Cocktail (Sigma-Aldrich) at 1/1,000 dilution, 0.2 mM PMSF, 1 mM benzamidine HCl, 1 mM DTT (Thermo Fisher Scientific), 1 mM NaN_3_, and 1% Triton X-100 to resuspend the tissue. The membrane permeabilization (skinning) was carried out on a rocker overnight at 4°C. The next morning, detergents were washed out by three changes of 15 ml of fresh, ice-cold relaxing buffer after the tissue was allowed to settle for 2 min. Tissue in relaxing buffer was transferred using a pipette with a wide bore tip into several glass tubes (12 × 35 mm; Thermo Fisher Scientific) for homogenization by a 5-mm homogenizer probe (Omni International) with three pulses of 10 s each at 20,000 rpm (position 4). Homogenization in small volumes (batches) increased homogeneity of the myofibril preparations. Myofibrils from each tube were then passed through 200-μm nylon mesh placed on a 15-ml Dounce homogenizer tube to remove remaining chunks of tissue. The combined filtrate was homogenized in a Dounce tube by five pumps using the B pestle. The resulting suspension was diluted with relaxing buffer to a final myofibrillar concentration of ∼1 mg/ml. Finally, myofibrils were centrifuged at 1,000 × *g* for 5 min to remove the mitochondrial supernatant. Typically, ∼500 mg of wet-frozen ventricle tissue resulted in 16 pellets of myofibrils, each ∼12 mg of wet mass (192 mg total) or ∼1 mg dry protein mass.

### Exchange of labeled troponin

Human recombinant IANBD-Tn was exchanged for endogenous porcine Tn in permeabilized myofibrils by a 4-h incubation on ice. Briefly, ∼48 mg of wet myofibrils (4 pellets of ∼12 mg each; or ∼4 mg of total protein, as ∼12 mg of wet myofibrils is ∼1 mg of protein mass) were resuspended and combined in a total of 200 μl of 23 ± 3 μM IANBD-labeled Tn in hypotonic rigor buffer containing of 6 mM ATP and 1 mg/ml BSA (to prevent clumping of myofibrils). The myofibril protein concentration during the exchange was ∼20 mg/ml. Following exchange, excess/non-exchanged Tn was removed by centrifuging myofibrils for 1,000 × *g* for 5 min and discarding the supernatant. Then, each myofibril pellet was resuspended in 1.2 ml of 0-EGTA rigor buffer (160 mM potassium propionate, 10 mM MOPS, 3 mM MgCl_2_, and 1 mg/ml BSA). The pellets were centrifuged/resuspended two more times before final resuspension in 0-EGTA rigor buffer (to ∼0.75 mg/ml myofibrillar protein). Lastly, the appropriate 10X Ca-EGTA buffer and drug (or 1% DMSO as a vehicle) was added to the myofibril suspension. Percent of exchanged Tn and total myofilament protein were determined by 18% SDS-PAGE of myofibrils and labeled-Tn standards dissolved in 7 M Tris-urea sample buffer (50 mM Tris, 7 M urea, and 2 mM EDTA, pH 7). After electrophoresis, fluorescence of labeled Tn in the gel was detected in a UV-box and followed by Coomassie staining of myofilament proteins.

### ATPase activity of myofibril preparations

To ensure that the Tn exchange does not perturb the normal function of myofibrils, ATPase activity was evaluated by a modified malachite green assay (Lu et al., 2010). Briefly, the myofibril pellet was resuspended in fresh 0-EGTA rigor buffer to 0.75 ± 0.15 mg protein/ml at 21°C in a water bath. Then, the myofibril suspension was split into two tubes for low and high Ca^2+^ conditions by adding 10× Ca^2+^-EGTA. Pre-warmed ATP was added to a final concentration of 0.1 mM to start the myosin ATPase reaction (*t* = 0 min). Every 1 or 2 min for 10–20 min, 10 μl of the reaction suspension was removed (out of 100 μl) and placed in the reaction stop solution (90 μl of ice-cold 0.2 M perchloric acid). 100 μl of malachite green solution was added to the 100 μl reaction, gently vortexed, and allowed to incubate for 10 min at room temperature (23°C) on a shaker. The malachite green solution was prepared by dissolving 2 g of ammonium molybdate (Sigma-Aldrich) in 700 ml of 1 M HCl, followed by the addition of 0.3 g of malachite green oxalate (Sigma-Aldrich), 5 ml of 10% Triton X-100 (Thermo Fisher Scientific), and double distilled H_2_O to a final volume of 1 liter. To determine the concentration of P_i_, absorbance was measured on a BioTek plate reader at 655 nm. Total protein concentration of myofibril samples was obtained by the BCA Protein Assay (Pierce, Thermo Fisher Scientific) or a Coomassie-stained SDS-PAGE. Results were expressed as nmol P_i_ per mg of protein per min (P_i_·mg^−1^·min^−1^), calculated using a P_i_ standard curve using NaH_2_PO_4_ standards. Three technical replicates were performed for high and low Ca^2+^ and measured in the presence of 1% DMSO or 100 μM Mava in DMSO.

### TR-F data acquisition

For TR-F experiments, 50 μl myofibrils (total protein 0.75 ± 0.15 mg/ml) were loaded into wells of 384-well flat, black-bottom polypropylene plates (Greiner Bio-One) using a P200 pipette and regular tips. The plate was sealed with a sheet of plate tape sealant and spun down at 1,000 rpm for ∼10 s to remove bubbles. In cases of high variability of fluorescence intensity, due to non-homogeneity of the myofibrillar suspension, several scans with different laser intensities were carried out to make sure that lifetimes were measured and compared under similar intensity levels. No photobleaching was observed as a result of repeated scans. Fluorescence lifetime measurements were acquired at room temperature (∼23°C) using a high-precision FLTPR (Fluorescence Innovations, Inc.; Schaaf et al., 2017; Bunch et al., 2018). The photomultiplier tube (PMT) voltage was adjusted so that the peak signals of the instrument response function (IRF) and the myofibril sample preparations had similar peak intensities (attenuated by a neutral density filter wheel; NDC-50C-2M, Thorlabs). The full nanosecond-resolved fluorescence emission waveform is acquired following excitation with a 473-nm pulsed microchip laser. The data acquisition time was ∼0.5 s (typically 200–500 ms) per well or ∼3 min per 384-well plate. The rapid data acquisition rate is made possible using direct wave recording (DWR) as described previously (Muretta et al., 2010). Fluorescence decay waveforms for lifetime determination were detected as previously described (Gruber et al., 2014; Petersen et al., 2014). IANBD was excited with a 473-nm passively Q-switched microchip laser (Bright Solutions) which delivers highly reproducible and high-energy pulses (∼1 μJ) at a 5-kHz repetition rate. Emission was filtered with a 488-nm long pass and 517/20-nm band pass filter (Semrock). A full fluorescence decay waveform was detected in response to each laser pulse. The PMT and digitizer are described previously (Petersen et al., 2014), but this FLTPR instrument employs epi-illumination excitation and detection from above.

### Ca^2+^ effects on TR-F

For cTnC^T53C^-IANBD–exchanged myofibrils, rigor buffer contained 0 mM EGTA, while 10× Ca^2+^-EGTA buffer contained 20 mM EGTA (final concentration of 2 mM). pCa (−log [Ca^2+^]) solutions tested were high calcium (pCa 4.5) and low calcium (pCa 9). Intermediate [Ca^2+^] of 7.4, 7, 6.6, and 6 were also tested. Concentrations of free Ca^2+^ were calculated by the WEBMAXC STANDARD resource (https://somapp.ucdmc.ucdavis.edu/pharmacology/bers/maxchelator/webmaxc/webmaxcS.htm).

### ATP effects on TR-F

After the first scan (“rigor” condition), we added 3 μl of ATP (Cat. No. BP413-25) to each well to a final concentration of 0.1 mM ATP using a multi-channel pipette. Plates were read at 20 min after addition of the nucleotide.

### Drug effects on TR-F

W7 hydrochloride (Bio-Techne), Pimo (Sigma-Aldrich), Mava (MedChemExpress), or OM (MedChemExpress) were added to the myofibrils in rigor buffer to a final concentration of 100 μM (from 10 mM stocks in DMSO). All compounds were tested for fluorescence, at the concentrations used in each experiment. Background fluorescence of all compounds did not exceed 5% threshold of the signal intensity. The final concentration of DMSO was 1% and no drug controls also contained 1% DMSO. Drugs were added to a tube of myofibrils and then pipetted into the microplate wells. Plates were read before the addition of ATP and at 3–4 and 20 min following the addition of ATP (to a final concentration of 0.1 mM).

### Sample exclusion

Inhomogeneities in myofibrils can result in high-fluorescence intensity in some wells due to chunks of tissue or clusters of labeled proteins not specifically bound to cardiac muscle tissues. These bright clusters/chunks can be seen by epifluorescence microscopy (data not shown). Wells with intensities above 200% of average or below 50%, due to pipetting flaws/air bubbles, were excluded. The percentage of samples in wells typically rejected based on aggregates was ∼3%.

### TR-F data analysis

Following data acquisition, TR-F waveforms observed for each well were analyzed using least squares minimization global analysis software (Petersen et al., 2014; Bunch et al., 2021a) and fitted (Eq. 1) by a simulation *S*_*D*_*(t)*, consisting of single-exponential decay model *M*_*D*_*(t)*, characterized by pre-exponential factor *A* and lifetime τ, convolved with the *IRF(t)*, acquired by recording scatter from the same volume double distilled H_2_O in a well of the 384-well plate.

The decay of the IANBD fluorescence was calculated as:

$$M_{D}\left(t\right)=A\exp\left(-t/\tau \right)\text{,}$$

$$S_{D}\left(t\right)=IRF\left(t-t^{\prime }\right)M_{D}\left(t^{\prime }\right)dt^{\prime }\text{.}$$ (1)

### Z′ factor analysis

The cTnC^T53C^-IANBD TR-F assay quality, for use in HTS, was determined by calculating the *Z*′ factor. This compares the average lifetimes and SD values of two states measured multiple times. Here, the two states include low versus high Ca^2+^; rigor versus ATP (or ADP) conditions; 1% DMSO versus drug (W7, Pimo, Mava, or OM) plus 1% DMSO. The *Z*′ factor was calculated as

$$Z^{\prime }=1‒3\left(\frac{\sigma_{1}+\sigma_{2}}{\left|\mu_{1}-\mu_{2}\right|}\right)\text{,}$$ (2)

where σ_1_ and σ_2_ are the SDs and μ_1_ and μ_2_ are the averages of the lifetimes (τ_1_ and τ_2_) from multiple samples of the two states. A value of 0–0.5 indicates feasible assay quality and 0.5–1 indicates excellent assay quality (Zhang et al., 1999; Rebbeck et al., 2017; Bunch et al., 2021b). In other words, *Z′* is positive (0 < *Z′* < 1) when the difference between the average signals (i.e., intensity or lifetime) of two conditions (e.g., low versus high Ca^2+^) is separated by more than three times SDs for each condition and *Z′* is negative (*Z′* < 0) when there is no separation (i.e., overlap) between three times SDs for each condition.

### Statistics

Average data are provided as mean ± SD. We performed four sets of myofibril experiments (separately exchanged preparations) from three separate batches of Tn. A two-tailed *t* test was used to make comparisons between two independent groups. A one-way ANOVA using Dunnett’s post-hoc test was used for comparisons of three groups (for bar graphs in figures a multiplicity adjusted P value was computed using GraphPad Prism version 9, GraphPad Software). P < 0.05 was considered significant. For each experiment, *n* = technical replicates or the number of wells containing myofibrils from a single mixture containing either high or low Ca^2+^ or DMSO versus drugs. Each *n* corresponds to one reading per well. Nucleotide was added to each well so that each well represents an independent response to the nucleotide. N = biological repeats or the number of independent purifications of recombinant proteins and/or preparations of skinned myofibrils.

### Online supplemental material

**Figure S1.**
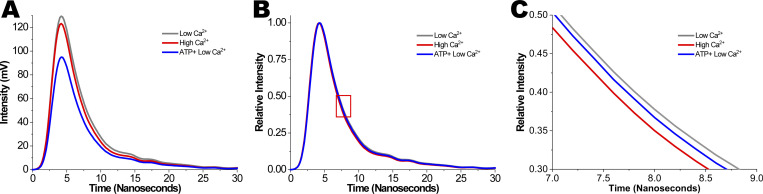
**Effects of Ca**^**2+**^ **and ATP on fluorescence intensity and lifetime in IANBD-cTnC**^**T53C**^ **exchanged myofibrils. (A)** Fluorescence waveforms of IANBD-cTnC^T53C^ exchanged myofibrils illustrate differences in intensities (peaks) due to Ca^2+^ and ATP. **(B)** Normalized fluorescence waveforms of IANBD-cTnC^T53C^ exchanged myofibrils allow for visual comparison of lifetimes (τ = time to decay to 1/e) due to Ca^2+^ and ATP. The red box highlights the waveform’s region of τ, which is zoomed-in on in C. **(C)** Zoom-in of red box in B shows separation of lifetime decays. Data is representative of Fig. 2.

**Figure S2.**
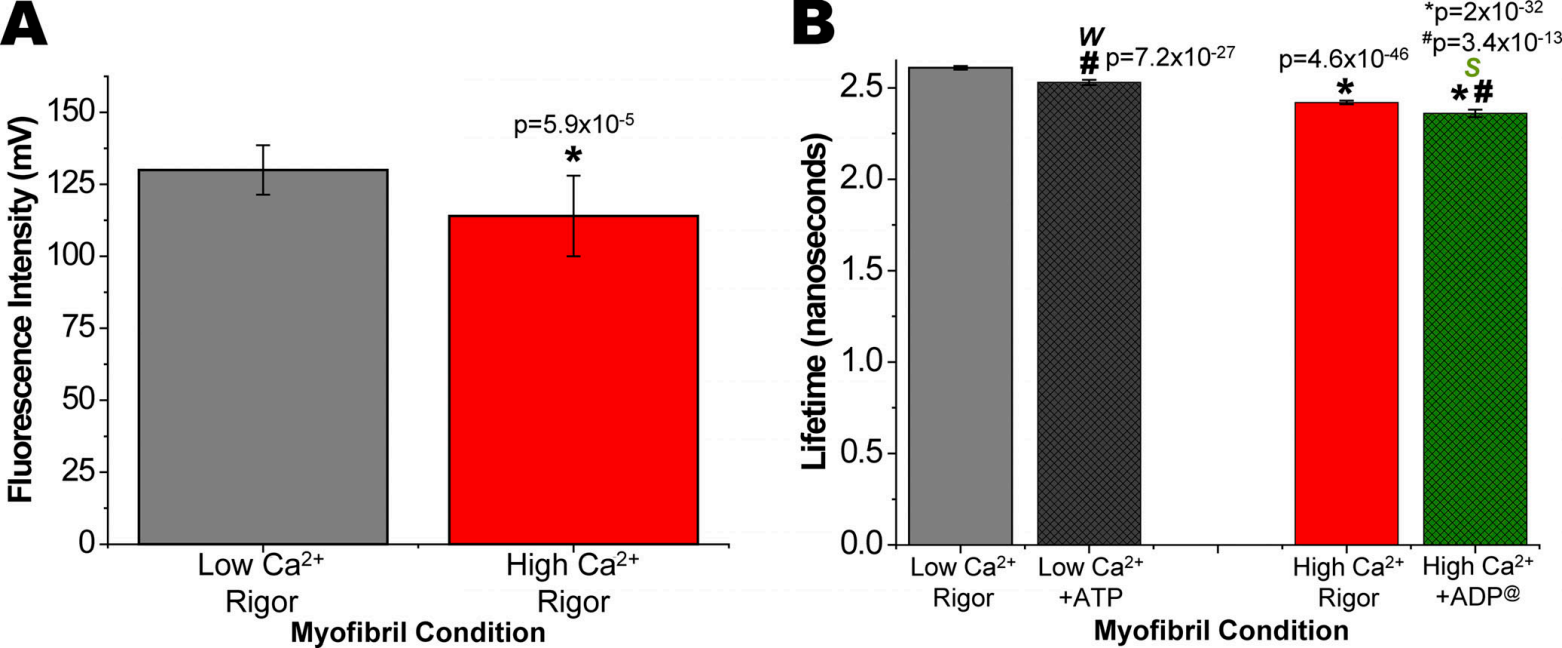
**Effects of Ca**^**2+**^ **and ATP on fluorescence intensity and lifetime in IANBD-cTnC**^**T53C**^ **exchanged myofibrils. (A and B)** Zoomed-in A and B from Fig. 2 (see legend) are plotted here on y-axis starting at (A) 0 mV (N = 1, *n* = 23) and (B) ns (N = 1, *n* = 23).

**Figure S3.**
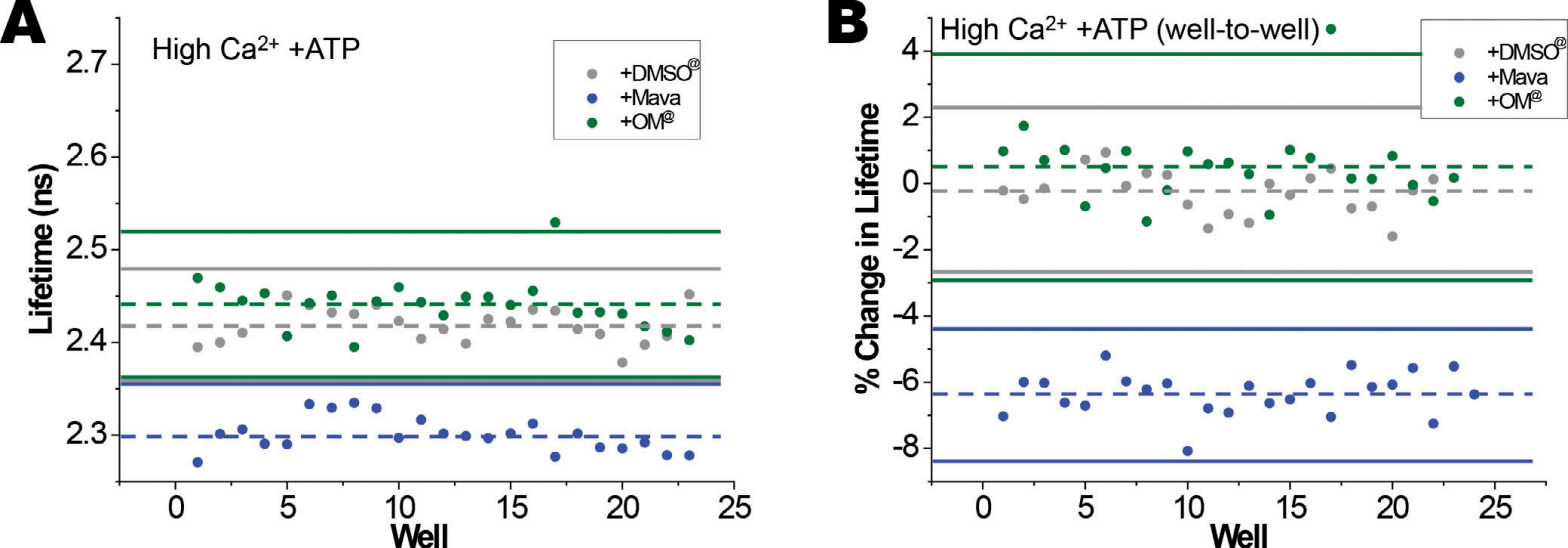
**Effects of Mava and OM on fluorescence lifetime in IANBD-cTnC**^**T53C**^ **exchanged myofibrils in a mock screen illustrates *Z′* quality of high-throughput assays. (A)** In the presence of ATP at high Ca^2+^, Mava reduces lifetime as compared to DMSO control (blue vs. grey: *Z′* = 0.04). For OM, 3× SD of the OM data overlap with 3× SD of the DMSO data (green vs. grey: *Z′* = −5.6). **(B)** When lifetime changes are corrected for well-to-well variability, and percentage change in lifetime following the addition of ATP, Mava effects are further separated from DMSO control (blue vs. grey: *Z′* = 0.27). In the mock screens (*n* = 23–24 wells for each of two conditions in A and B), the average for each condition is indicated with a dashed line and 3× SD above and below the average is shown with a solid line. ^@^With high Ca^2+^ in control and +OM at 20 min, ATP has been hydrolyzed to ADP.

The supplemental material contains results of representative fluorescence waveforms, changes in intensities and lifetimes zoomed-out and plotted from zero for reference, and additional conditions (ATP, Ca^2+^) of mock screens using the four drugs. Fig. S1 shows the effects of Ca^2+^ and ATP on fluorescence intensity and lifetime in IANBD-cTnC^T53C^ exchanged myofibrils. Fig S2 shows the effects of Ca^2+^ and ATP on fluorescence intensity and lifetime in IANBD-cTnC^T53C^ exchanged myofibrils. Fig. S3 shows the effects of Mava and OM on fluorescence lifetime in IANBD-cTnC^T53C^ exchanged myofibrils in a mock screen illustrates *Z*′ quality of high-throughput assays. Table S1 provides IANBD-cTnC^T53C^ fluorescence lifetime changes due to W7 and Pimo in low Ca^2+^. Table S2 provides IANBD-cTnC^T53C^ fluorescence lifetime changes due to W7 and Pimo in high Ca^2+^. Table S3 provides IANBD-cTnC^T53C^ fluorescence lifetime changes due to Mava and OM in low Ca^2+^. Table S4 provides IANBD-cTnC^T53C^ fluorescence lifetime changes due to Mava and OM in high Ca^2+^. Table S5 provides IANBD-cTnC^T53C^ fluorescence lifetime changes due to Mava and OM by well-to-well analysis at high Ca^2+^.

## Results

### Incorporation of IANBD-labeled cTnC^T53^ into porcine myocardium

**Figure 1.**
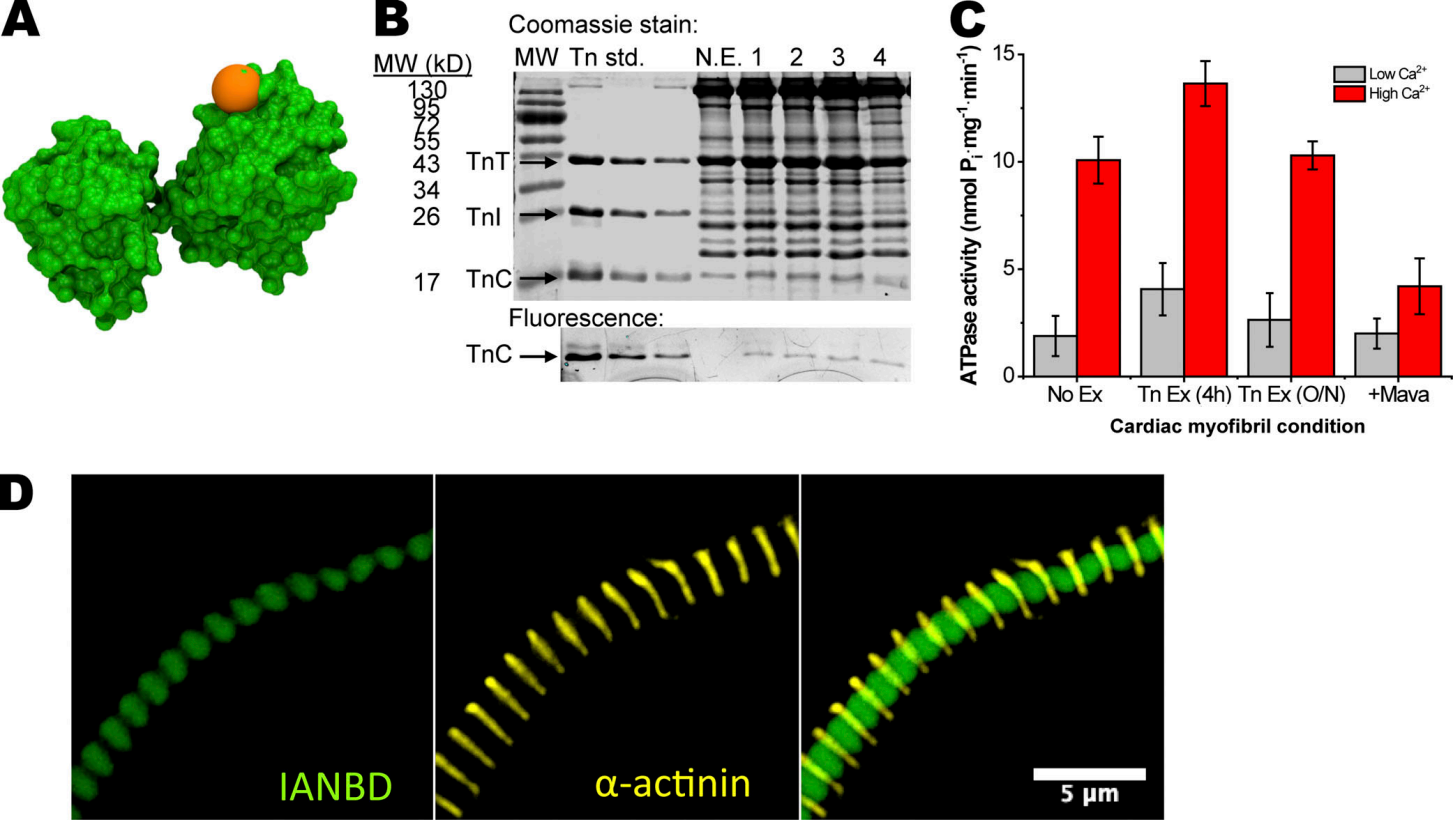
**IANBD-cTnC**^**T53C**^ **exchange into skinned cardiac porcine myofibrils.** IANBD-cTnC^T53C^ is properly incorporated into myofibrils without loss of function. **(A)** Structure of cTnC (PDB accession no. 4Y99). Orange ball represents the IANBD probe covalently attached to residue 53. **(B)** 18% SDS-PAGE gel showing: molecular weight markers (MW, lane 1), dilutions of Tn containing IANBD-cTnC^T53C^ used for exchanges (Tn standard, lanes 2–4), non-exchanged myofibrils (N.E., lane 5), and four separate exchanges of Tn containing IANBD-cTnC^T53C^ into myofibrils (1–4, lanes 6–10). The top image (Coomassie stain) shows the gel stained with Coomassie Blue to visualize total protein. The bottom image (fluorescence) is the region of the gel containing cTnC imaged for fluorescence signal. The level of IANBD-cTnC^T53C^ incorporation was calculated using ratios of the labeled protein IANBD-cTnC^T53C^ (bottom gel: fluorescent cTnC) to total cTnC (top gel: cTnC). Specifically, troponin (Tn) with IANBD-TnC^T53C^, prior to exchange, was used to determine the fluorescence/Coomassie ratio for cTnC in the input labeled Tn (Tn standard lanes). Ratios of fluorescence/Coomassie intensities of cTnC for 4 (1–4) exchanges were compared with the input labeled Tn to obtain the level of exchange. **(C)** Myosin ATPase activity (nmol of P_i_ released·mg^−1^ of myofibrillar protein·min^−1^) at low and high Ca^2+^ was similar for no exchange (No Ex., i.e., mock incubation) and Tn exchange (Tn Ex., i.e., containing IANBD-cTnC^T53C^) for 4 h or overnight (O/N) incubations. Following exchange (4 h), 100 μM Mava (+Mava) treatment reduced ATPase activity. N = 2, *n* = 6. **(D)** Fluorescence microscopy images of IANBD-cTnC^T53C^–exchanged myofibrils (100× objective). Image of a single myofibril with filters showing IANBD-TnC^T53C^ (green), α-actinin antibody (yellow), and overlay show proper localization of exchanged IANBD-cTnC^T53C^. Source data are available for this figure: SourceData F1.

We reconstituted IANBD-labeled cTnC^T53C^ (Fig. 1 A) into cardiac troponin (combined with wild-type human cTnI and cTnT) and exchanged this for endogenous cardiac troponin in membrane-permeabilized (skinned) porcine ventricular myofibrils by mass action exchange (Dvornikov et al., 2016). The estimated percentage of exchange in the myofibrils was determined by the fluorescence signal (IANBD) of labeled cTnC, relative to total cTnC on Coomassie-stained gels. The extent of exchange in our experiments was 41 ± 9%, which did not differ between 4 h and overnight exchange times (Fig. 1 B). Similarly, myofibrillar ATPase function was not significantly affected by the exchange procedure (Fig. 1 C; see also next section). Fluorescence microscopy showed incorporation of the IANBD-cTnC^T53C^ between Z-discs (stained with α-actinin antibodies) suggesting the incorporation of IANBD-Tn onto the thin filaments between Z-discs (Fig. 1 D).

### Function of IANBD-cTnC^T53C^-exchanged myofibril preparations

Myofibrils exchanged with IANBD-cTnC^T53C^ demonstrated normal function as measured by their myosin ATPase activity. Basal ATPase activity in low Ca^2+^ (pCa 9: ∼2–4 nmol P_i_·mg^−1^·min^−1^) and maximal ATPase activity in high Ca^2+^ (pCa 4.5: ∼11–14 nmol P_i_·mg^−1^·min^−1^) were not different at each respective Ca^2+^ level between non-exchanged and exchanged myofibrils with 0.1 mM ATP (Fig. 1 C). At these ATPase rates, 0.1 mM ATP at pCa 9 is consumed after ∼60–110 and ∼14–19 min at pCa 4.5. Therefore, for TR-F experiments, ATP was present for 0.1 mM ATP at 20 min for low Ca^2+^ conditions (i.e., weakly bound myosin). ADP was present at 20 min in high Ca^2+^ conditions (i.e., strongly bound myosin). Following exchange, addition of 100 μM Mava reduced myofibril ATPase to ∼2 nmol P_i_·mg^−1^·min^−1^ in low Ca^2+^ and to ∼4 nmol P_i_·mg^−1^·min^−1^ in high Ca^2+^ (Fig. 1 C). In the presence of Mava, it would take ∼94 and ∼45 min to hydrolyze ATP to ADP in low and high Ca^2+^, respectively. Thus, when Mava is present, ATP was the predominant nucleotide present in both low and high Ca^2+^ at 20 min.

### IANBD-cTnC^T53C^ lifetimes report changes due to Ca^2+^ that are sufficient for use in HTS

**Figure 2.**
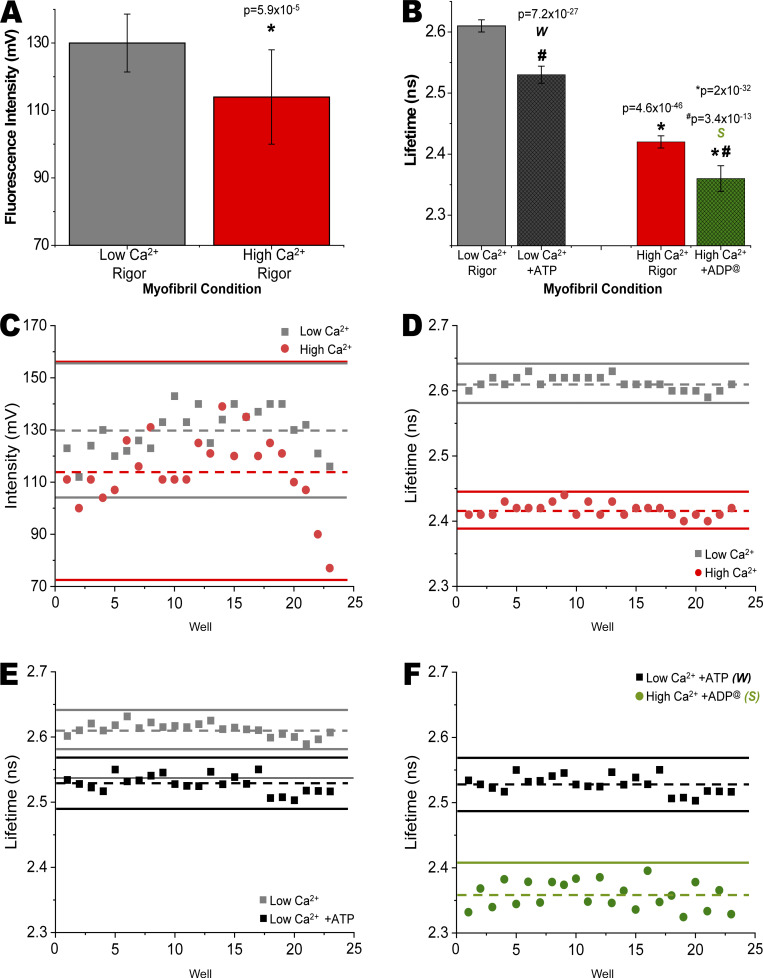
**Effects of Ca**^**2+**^ **and ATP on fluorescence intensity and lifetime in IANBD-cTnC**^**T53C**^ **exchanged myofibrils. (A)** In rigor conditions, IANBD-cTnC intensity in myofibrils is reduced by high Ca^2+^ (red) compared with low Ca^2+^ (grey; *P = 5.9 × 10^−5^). *n* = 23 repeats in wells from N = 1 experimental preparation. **(B)** In rigor conditions (grey and red), IANBD-cTnC lifetime is reduced by Ca^2+^ (*P = 4.6 × 10^−46^). Effects of ATP, releasing the rigor bound myosin from actin, at low Ca^2+^ were significant (black vs. grey; ^#^P = 7.2 × 10^−27^). In the presence of ATP, the effects of Ca^2+^ were clear. The difference between low Ca^2+^ +ATP (black, weak-binding myosin [*W*]) and high Ca^2+^ +ADP^@^, i.e., 0.1 mM ATP hydrolyzed in pCa 4.5 after 20 min (green, strong-binding myosin [*S*]) was very significant (*P = 2 × 10^−32^). **(C–F)** In the mock screens (∼24 wells for each of two conditions in C–F) the average for each condition is indicated with a dashed line and 3× SD above and below the average is shown with a solid line. *n* = 23 repeats in wells from N = 1 experimental preparation. Shown are Ca^2+^ effects on (C) intensity (*Z′* = −3.45) and (D) lifetime (*Z′* = 0.70), (E) ATP effects on lifetime at low Ca^2+^ (*Z′* = 0.16) and (F) weak (*W*; Ca^2+^ +ATP) versus strong (*S*; high Ca^2+^ +ADP^@^)–binding myosin (*Z′* = 0.39; see Table 3).

In rigor conditions, Ca^2+^ binding to cTnC decreased the intensity and lifetime of IANBD attached to residue 53 in cTnC^T53C^ incorporated into myofibrils by 9.5 and 7.5%, respectively (Fig. 2, A and B). At low and high Ca^2+^ concentrations, absolute waveforms demonstrate changes in intensity and normalized waveforms show changes in lifetime for IANBD-cTnC^T53C^ myofibrils (Fig. S1). Versions of the bar graphs in Fig. 2, A and B, with y-axis scales starting at 0 mV or ns are shown for reference in Fig. S2. The IANBD intensity is concentration-dependent and small differences, likely due to pipetting of exchanged myofibrils, account for the observed SD of ∼9 mV and coefficient of variance (C.V.) of ∼7%. While changes in intensity were significant (P = 1 × 10^−4^), the relatively large well-to-well variations result in a negative Z-factor (*Z*′; −3.6) that precludes its use in HTS designed to identify compounds that modify troponin’s response to Ca^2+^ (Zhang et al., 1999; Fig. 2 C).

**Figure 3.**
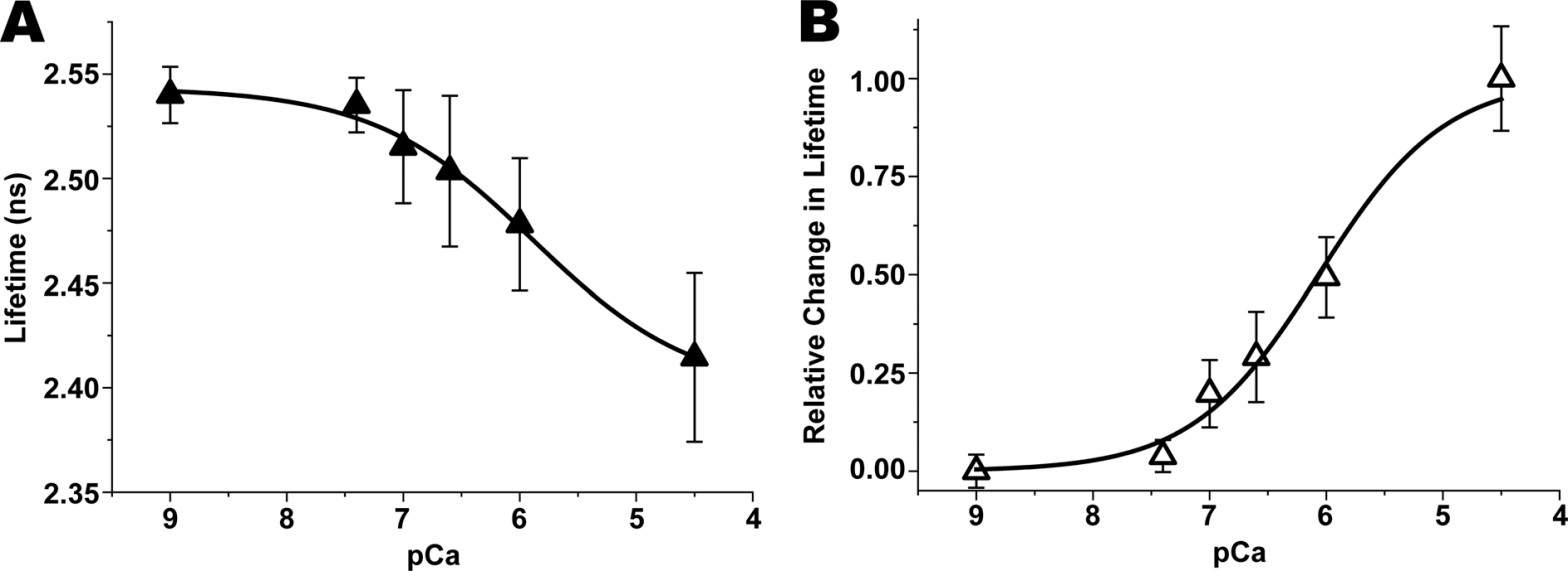
**Effects of Ca**^**2+**^ **concentration on lifetime in IANBD-cTnC**^**T53C**^ **exchanged myofibrils. (A)** Decreases in lifetime (ns) with increasing [Ca^2+^] (i.e., sigmoidal dose-response plot of [Ca^2+^] on lifetime) in solid triangles. **(B)** Relative change in lifetime with increasing [Ca^2+^] (i.e., relative lifetime-pCa plot) with pCa 9.0 at 0 and pCa 4.5 normalized to 1 in open triangles. Error bars are SE. For B, fit to a Hill equation, pCa_50_ was 6.07 ± 0.08 and the Hill coefficient η was 0.8 ± 0.1. N = 4, *n* = 16.

Changes in lifetime due to Ca^2+^ binding were far more significant (P = 4.6 × 10^−46^) compared to intensity, and with low well-to-well variability (reflected in a greatly reduced SD of ∼0.01 ns and C.V. of ∼0.4%), thereby offering a suitable assay for use in HTS. This was demonstrated by determining a Z-factor (*Z*′) and is illustrated in Fig. 2 D. There is clearly no overlap of 3× SDs of low and high Ca^2+^ and the *Z*′ of 0.7 indicates excellent conditions for high throughput screening. Repeating this experiment of comparing intensity and lifetime changes of IANBD-cTnC^T53C^ four times with three independent preparations of IANBD-Tn and myofibrils gave similar results (Tables 1 and 2). For all of the following experiments, we focus only on lifetime changes due to its superior precision. As the myocardium operates over a wide range of [Ca^2+^], we also examined effects of four intermediate physiological [Ca^2+^] levels: pCa 7.4, 7, 6.6, and 6. Lifetime-pCa plots are shown in Fig. 3 with a pCa_50_ value of 6.07 ± 0.08 and Hill coefficient η of 0.8 ± 0.1.

**Table 1.** IANBD-cTnC^T53C^ fluorescence intensity changes with low and high Ca^2+^

Experiment (N)	Buffer condition	Buffer condition	Average intensity	SD	C.V.	*n*	Change + Ca^2+^	*Z*′	P
#1	Rigor	Low Ca^2+^	130	9	6.6%	23			
	Rigor	High Ca^2+^	117	10	8.9%	21	−9.5%	−3.6	1.0 × 10^-4^
#2	Rigor	Low Ca^2+^	129	17	13.4%	24			
	Rigor	High Ca^2+^	123	16	13.4%	23	−4.8%	−15.3	0.215
#3	Rigor	Low Ca^2+^	135	26	19.3%	23			
	Rigor	High Ca^2+^	111	27	24.2%	23	−17.5%	−5.7	0.004
#4	Rigor	Low Ca^2+^	141	7	5.2%	12			
	Rigor	High Ca^2+^	129	16	12.5%	12	−8.1%	−5.2	0.036
Average	Rigor	Low Ca^2+^	−	−	−				
	Rigor	High Ca^2+^	−	−	−		−10.0%	−7.5	0.06

Average data are provided for individual experiments. Experiments were carried out with three separate protein preparations of troponin that was exchanged into four separate myofibril preparations. Low Ca^2+^ is pCa 9 and High Ca^2+^ is pCa 4.5. The unit for Average (fluorescence) intensity of IANBD-cTnC^T53C^ and SD is millivolts. *n* = number of wells of myofibrils into which low or high Ca^2+^ is individually added. Change + Ca^2+^ is the percentage change in intensity between low and high Ca^2+^ in each experiment.  C.V. is the coefficient of variance. Statistical tests of *Z′* factor and *t* test are used to evaluate the change in intensity between low and high Ca^2+^. The average *Z′* and percentage Change + Ca^2+^ for the four experiments is also given.

**Table 2.** IANBD-cTnC^T53C^ fluorescence lifetime changes with low and high Ca^2+^

Experiment (N)	Buffer condition	Buffer condition	Average lifetime	SD	C.V.	*n*	Change + Ca^2+^	*Z*′	P
#1	Rigor	Low Ca^2+^	2.61	0.01	0.4%	23			
	Rigor	High Ca^2+^	2.42	0.02	0.4%	23	−7.5%	0.70	4.6 × 10^−46^
#2	Rigor	Low Ca^2+^	2.67	0.02	0.7%	23			
	Rigor	High Ca^2+^	2.42	0.01	0.5%	23	−9.4%	0.64	3.2 × 10^−42^
#3	Rigor	Low Ca^2+^	2.69	0.01	0.5%	24			
	Rigor	High Ca^2+^	2.39	0.02	0.8%	24	−11.1%	0.65	4.1 × 10^−45^
#4	Rigor	Low Ca^2+^	2.62	0.01	0.4%	12			
	Rigor	High Ca^2+^	2.37	0.02	0.7%	12	−9.6%	0.68	3.1 × 10^−23^
Average	Rigor	Low Ca^2+^	−	−	−				
	Rigor	High Ca^2+^	−	−	−		−9.4%	0.67	7.7 × 10^−24^

Average data are provided for individual experiments. Experiments were carried out with three separate protein preparations of troponin that was exchanged into four separate myofibril preparations. Low Ca^2+^ is pCa 9 and High Ca^2+^ is pCa 4.5. The unit for Average (fluorescence) lifetime and SD (standard deviation) is nanoseconds. *n* = number of wells of myofibrils into which low or high Ca^2+^ is individually added. Change + Ca^2+^ is the percentage change in lifetime between low and high Ca^2+^ in each experiment. C.V. is the coefficient of variance. Statistical tests of *Z′* factor and *t* test are used to evaluate the change in lifetime between low and high Ca^2+^. The average *Z′* and percentage Change + Ca^2+^ for the four experiments is also given.

The comparisons of low, intermediate, and high [Ca^2+^] were performed in rigor conditions so that there would not be differences in troponin due to the effects of myosin binding to actin. In rigor cardiac muscle, myosin is strongly bound to the thin filament in all Ca^2+^ conditions. Addition of nucleotide (ATP) complicates examination of Ca^2+^ effects due to variable myosin binding (see next section). Despite this complication we observed that Ca^2+^ caused a reduction in the lifetime of IANBD on cTnC^T53C^ in the presence of nucleotide (see also Fig. S1). Comparing low and high Ca^2+^ conditions in the presence of 0.1 mM ATP (initial concentration), the IANBD lifetime was reduced by 6.7% (P = 2.3 × 10^-32^; Fig. 2 B). The *Z*′ for this experiment (*Z*′ = 0.39, Fig. 2 F) and the average *Z*′ for four experiments (average *Z*′ = 0.32, Table 3) indicated that conditions are good for HTS of compounds affecting Ca^2+^ binding by cTnC. In these conditions, 20 min after the addition of 0.1 mM ATP, at low Ca^2+^, most (65–80%) of the ATP remains unhydrolyzed and myosin only weakly binds actin. At high Ca^2+^, by 20 min, myosin has hydrolyzed the ATP to ADP (see previous section and Fig. 1 C), resulting in strongly bound myosin-ADP that increases the lifetime of IANBD on cTnC^T53C^ as explored in the next section. Despite the increase in lifetime due to myosin binding, at high Ca^2+^ we still observe an overall decrease in lifetime due to the effect of Ca^2+^ binding to cTnC.

**Table 3.** IANBD-cTnC^T53C^ fluorescence lifetime changes with myosin in weakly and strongly bound states

Experiment (N)	Buffer condition*	Myosin-binding	Average lifetime	SD	C.V.	*n*	Change + Ca^2+^	*Z*′	P
#1	ATP, low Ca^2+^	Weak	2.53	0.01	0.5%	23			
	^@^ADP, high Ca^2+^	Strong	2.36	0.02	0.9%	23	−6.7%	0.39	2.3 × 10^−32^
#2	ATP, low Ca^2+^	Weak	2.61	0.01	0.5%	23			
	^@^ADP, high Ca^2+^	Strong	2.42	0.02	0.8%	22	−7.2%	0.47	1.2 × 10^−34^
#3	ATP, low Ca^2+^	Weak	2.56	0.02	0.7%	24			
	^@^ADP, high Ca^2+^	Strong	2.28	0.03	1.4%	24	−10.8%	0.44	3.1 × 10^−35^
#4	ATP, low Ca^2+^	Weak	2.56	0.04	1.5%	12			
	^@^ADP, high Ca^2+^	Strong	2.36	0.03	1.4%	12	−8.2%	−0.02	1.4 × 10^−12^
Avg	ATP, low Ca^2+^	Weak	2.56	−	−				
	^@^ADP, high Ca^2+^	Strong	2.35	−	−		−8.2%	0.32	3.4 × 10^−13^

Average data are provided for individual experiments. Experiments were carried out with three separate protein preparations of troponin that was exchanged into four separate myofibril preparations. Low Ca^2+^ is pCa 9 and High Ca^2+^ is pCa 4.5. The unit for Average (fluorescence) lifetime and SD (standard deviation) is nanoseconds. *n* = number of wells of myofibrils (in rigor buffer) into which ATP and low or high Ca^2+^ is individually added and scanned at 20 min, following an initial scan in rigor buffer (Change + Ca^2+^). C.V. is the coefficient of variance. Statistical tests of *Z′* factor and *t* test are used to evaluate the change in lifetime between low and high Ca^2+^ in the presence of ATP. ^@^ADP, high Ca^2+^ buffer condition indicates that 0.1 mM ATP was added in pCa 4.5 and after 20 min, all ATP was hydrolyzed to ADP due to ATPase activity of myofibrils (see Fig. 1 C and Function of IANBD-cTnC^T53C^-exchanged myofibril preparations). The average *Z′* and percentage Changes from Weak to Strong myosin-binding for the four experiments is also given.

### cTnC^T53C^-IANBD lifetime reports changes due to myosin binding the thin filament that are large enough for use in HTS

Myosin binding to the thin filament in rigor conditions increased the lifetime of IANBD on cTnC^T53C^ when compared to binding in the presence of nucleotide (Fig. 2, B and E; and Fig. S2) at either low or high Ca^2+^. This is demonstrated by observing the cTnC^T53C^-IANBD lifetime in low Ca^2+^ (pCa 9) and high Ca^2+^ (pCa 4.5) first in rigor conditions and then again 20 min after the addition of 0.1 mM ATP. In low Ca^2+^, the rigor bound-myosin showed a cTnC^T53C^-IANBD lifetime that was 3.3% greater than that seen when ATP was present (Fig. 2 B). This difference in lifetime of IANBD-cTnC was significant (P = 7.2 × 10^−27^) and large enough to be used in an HTS as shown by a positive *Z*′ (0.16) in this experiment (Fig. 2 E) and an average *Z*′ of 0.11 for four experiments (Table 4).

**Table 4.** IANBD-cTnC^T53C^ fluorescence lifetime changes upon myosin release from rigor with ATP in low Ca^2+^

Experiment (N)	Buffer condition	Buffer condition	Average lifetime	SD	C.V.	*n*	Change + ATP	*Z*′	P
#1	Rigor	Low Ca^2+^	2.61	0.01	0.4%	23			
	ATP	Low Ca^2+^	2.53	0.01	0.5%	23	−3.2%	0.16	7.2 × 10^−27^
#2	Rigor	Low Ca^2+^	2.67	0.02	0.7%	23			
	ATP	Low Ca^2+^	2.61	0.01	0.5%	23	−2.5%	−0.41	4.1 × 10^−18^
#3	Rigor	Low Ca^2+^	2.69	0.01	0.5%	24			
	ATP	Low Ca^2+^	2.56	0.02	0.7%	24	−4.8%	0.22	1.6 × 10^−29^
#4	Rigor	Low Ca^2+^	2.54	0.01	0.5%	11			
	ATP	Low Ca^2+^	2.41	0.01	0.4%	11	−5.2%	0.49	2.4 × 10^−17^
Average	Rigor	Low Ca^2+^	−	−	−				
	ATP	Low Ca^2+^	−	−	−		−3.9%	0.11	7.0 × 10^−18^

Average data are provided for individual experiments. Experiments were carried out with three separate protein preparations of troponin that was exchanged into four separate myofibril preparations. Low Ca^2+^ is pCa 9. The unit for Average (fluorescence) lifetime and SD is nanoseconds (ns). *n* = number of wells of myofibrils (in rigor buffer) into which low Ca^2+^ or low Ca^2+^ plus ATP is individually added and scanned at 20 min. Change + ATP is the percentage change in lifetime between low Ca^2+^ without nucleotide and low Ca^2+^ + ATP for each experiment. C.V. is the coefficient of variance. Statistical tests of *Z′* factor and *t* test are used to evaluate the change in lifetime between addition of equal volumes of rigor buffer and ATP in low Ca^2+^. The average *Z′* and percentage Change + ATP for the four experiments is also given.

### cTnC^T53C^-IANBD lifetime reports changes due to the Ca^2+^ desensitizer W7 and Ca^2+^-sensitizer Pimo

**Figure 4.**
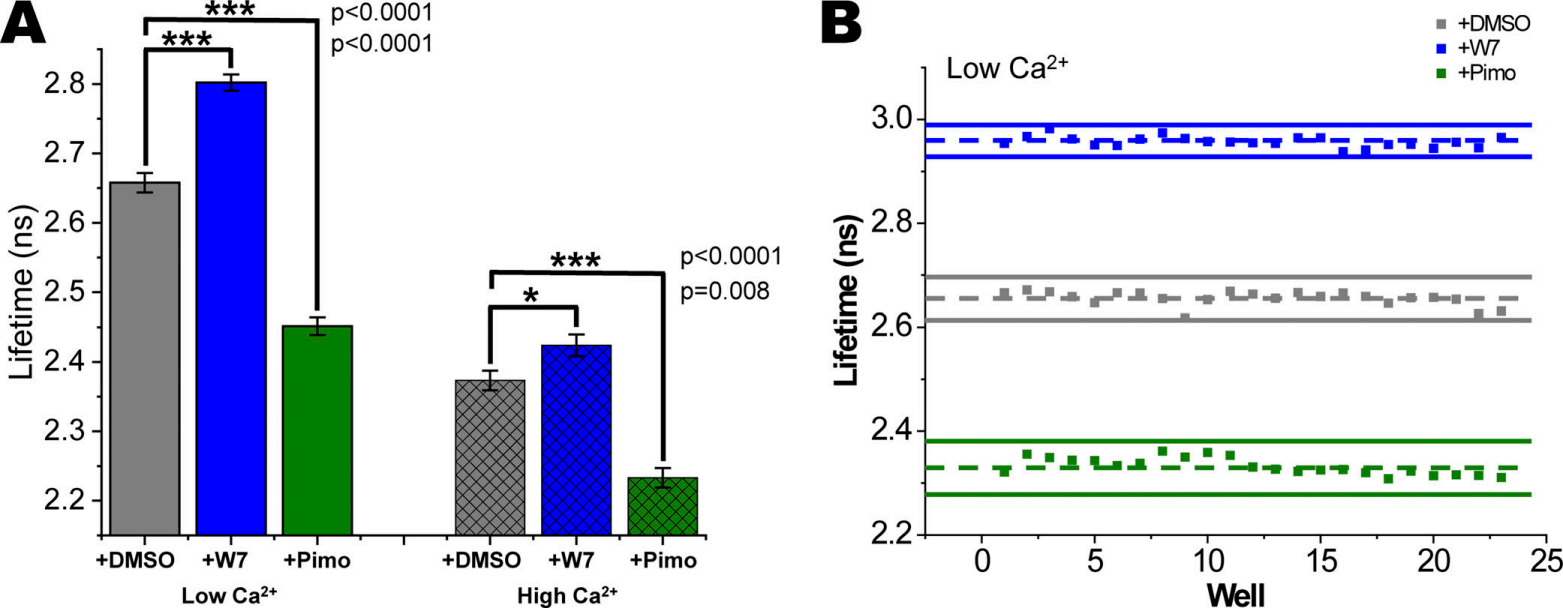
**Effects of W7 and Pimo on fluorescence lifetime in IANBD-cTnC**^**T53C**^ **exchanged myofibrils in a mock screen illustrates *Z′* quality of high-throughput assays. (A)** IANBD-cTnC lifetime is increased with W7 at low (left blue) and high Ca^2+^ (right hatched blue). Pimo causes a decrease in lifetime at low (left green) and high (right hatched green) Ca^2+^, ***P < 0.0001, *P < 0.01. Data is mean ± SD; W7 and Pimo data are averages of N = 2 experimental preparations, whereas DMSO (control) is the average of N = 7 experiments (i.e., DMSO data was combined from Mava and OM experiments); *n* = 48–140. **(B)** At low Ca^2+^, W7 increased lifetime as compared to DMSO control (blue vs. grey: *Z′* = 0.71) and effects of Pimo decreased lifetime as compared to DMSO (green vs. grey: *Z′* = 0.22). In the mock screens (*n* = 23 wells for each of two conditions) the average for each condition is indicated with a dashed line and 3× SD above and below the average is shown with a solid line.

In rigor conditions at low Ca^2+^ (pCa 9), W7 increased lifetime by 11.4% as compared to DMSO control (P = 3.1 × 10^−51^; Fig. 4 A). At high Ca^2+^ (pCa 4.5), W7 increased the lifetime by 6.1% relative to DMSO (P = 1.5 × 10^−33^). Moreover, the change in lifetime from low to high Ca^2+^ was 64% larger with W7 as compared to DMSO (Δτ of 0.39 ns versus 0.24 ns; P = 3.6 × 10^−29^). In a representative mock screen of 24 wells, the *Z*′ factor was 0.76 (blue vs. grey in Fig. 4 B), indicating an excellent assay for use in HTS. In two separate preparations of mock screens, the average *Z*′ factor was 0.73 for low Ca^2+^ (Table S1). *Z*′ factor was 0.37 for a representative mock screen at high Ca^2+^ (blue vs. grey in Fig. 4 C) and 0.30 for an average of two preparations (Table S2), indicating a good assay for use in HTS.

In contrast to W7, Pimo decreased the lifetime as compared to DMSO by 3.2% in low Ca^2+^ (P = 2.3 × 10^−28^) and 2.5% in high Ca^2+^ (P = 6.1 × 10^−19^; green vs. grey in Fig. 3, B and C). The change in lifetime between low and high Ca^2+^, however, was not different between Pimo and DMSO (Δτ of ∼0.21 ns for both). In one mock screen, *Z*′ factor was 0.22 at low Ca^2+^ (Fig. 4 B). Repeating this assay with a separate preparation again showed significant effects (P = 1.7 × 10^−6^) with a *Z*′ factor of −2.7 (Table S1). Combining these two experiments gives an average *Z*′ factor of −1.25, indicating that detecting a compound like Pimo in the current HTS assay conditions is not feasible (Table S1). At high Ca^2+^ (Fig. 4 C and Table S2), we found a significant change in the lifetime of cTnC^T53C^-IANBD when Pimo was added (P = 2.8 × 10^−15^ and P = 6.1 × 10^−19^) and again the average *Z*′ factor was negative (−0.58) indicating that the conditions are not useful for an HTS assay that would reliably detect Pimo.

### Effects of myosin-binding drugs Mava and OM

**Figure 5.**
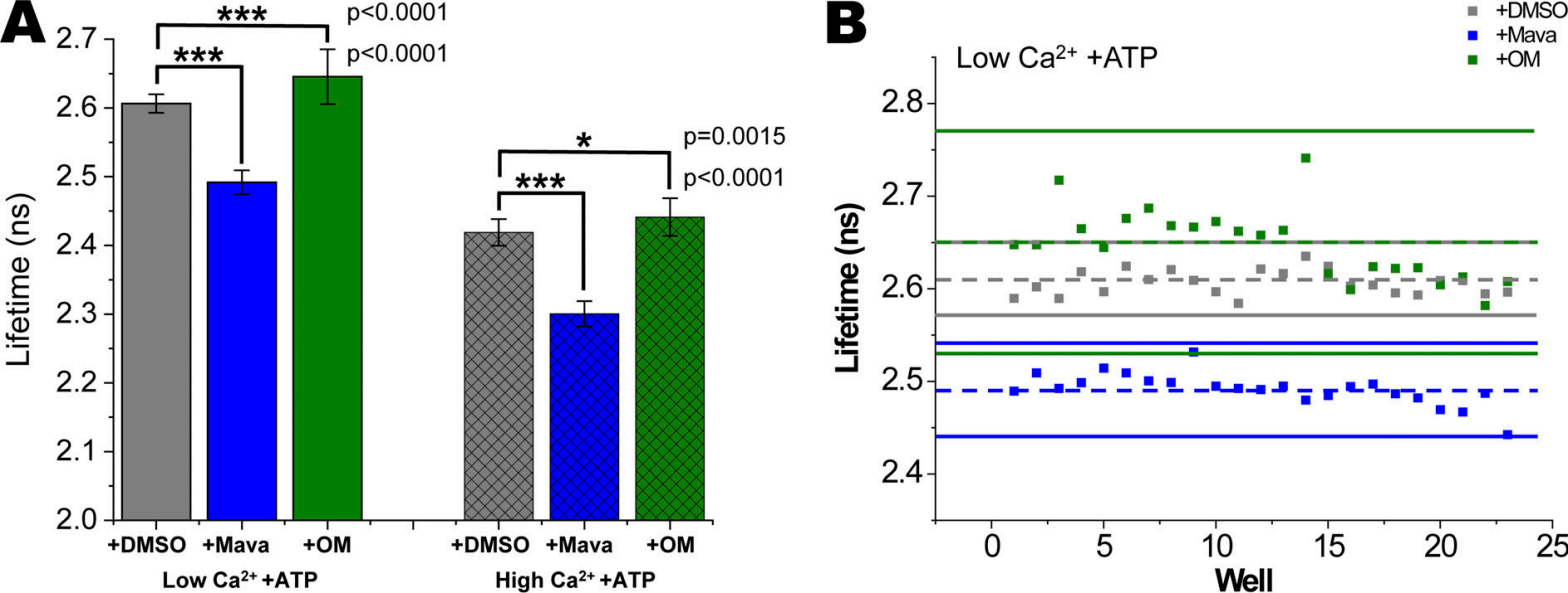
**Effects of Mava and OM on fluorescence lifetime in IANBD-cTnC**^**T53C**^ **exchanged myofibrils in a mock screen illustrates *Z′* quality of high-throughput assays. (A)** IANBD-cTnC lifetime is reduced with Mava in the presence of ATP at low (left) and high (right) Ca^2+^. OM causes small but significant increases in lifetime at low and high Ca^2+^, ***P < 0.0001, *P < 0.002. Data is mean ± SD; *n* = 23 repeats in wells from one experimental preparation. **(B)** In the presence of ATP at low Ca^2+^, Mava reduces lifetime as compared to DMSO control and 3× SD of the Mava data is well separated from 3× SD of the DMSO data (blue vs. grey: *Z′* = 0.19). For OM, 3× SD of the OM data overlap with 3× SD of the DMSO data (green vs. grey: *Z′* = −2.71). In the mock screen (*n* = 23 wells for each of two conditions) the average for each condition is indicated with a dashed line and 3× SD above and below the average is shown with a solid line. Note that dashed green (mean + OM) line is coincident with the upper solid grey line (+3× SD +DMSO).

The myosin inhibitor Mava reduced myosin effects on IANBD-cTnC lifetimes in both low and high Ca^2+^. At both low and high Ca^2+^, the addition of ATP to myofibrils in rigor reduced the lifetime of IANBD-cTnC (Fig. 2 B). The presence of Mava further significantly reduced the lifetime of IANBD-cTnC (Fig. 5 A). In low Ca^2+^ (pCa 9), with 0.1 mM ATP, the lifetime of IANBD-cTnC decreased from 2.61 ± 0.01 ns in the absence of Mava to 2.49 ± 0.02 ns in the presence of Mava. This 4.4% decrease in lifetime is highly significant (P = 1.6 × 10^−27^). *Z*′ for this experiment (Fig. 5 B; low Ca^2+^ ± Mava) was 0.19 and the average of three tests at low Ca^2+^ was 0.39 (Table S3). This indicates that screens for drugs having similar effects as Mava are attainable at low Ca^2+^.

At high Ca^2+^, with 0.1 mM ATP, the reduction in lifetime was from 2.35 ± 0.03 ns to 2.25 ± 0.02 ns upon the addition of Mava. This decrease of 4.3% was significant (P = 4.4 × 10^−10^). The average *Z*′ for four tests was −0.48, indicating that this test condition is not useful for an HTS assay at high Ca^2+^ (Fig. S3 A and Table S4). However, by paired well-to-well analysis, positive *Z*′ could be attained (see Fig. S3 B and Table S5), suggesting promise for a useful HTS assay at high Ca^2+^.

OM, a drug that promotes the ATP bound state of myosin, had a small but significant effect on IANBD-cTnC lifetime in both low and high Ca^2+^ when ATP is present (Fig. 5, Tables S3, S4, and S5). At low Ca^2+^ the average P = 3.9 × 10^−3^ and at a high Ca^2+^ average P = 2.1 × 10^−3^. These effects of increased lifetime were the opposite of that observed with Mava. In neither low (Fig. 5 B) nor high Ca^2+^ (Fig. S3) conditions were the changes in lifetime large and precise enough (average *Z*′ = −3.5 and −4.1, respectively) to be useful in HTS (Tables S3, S4, and S5).

## Discussion

In the present study, we demonstrate that lifetime measurements of fluorescent IANBD-cTnC incorporated into cardiac myofibrils can sense Ca^2+^ binding to cTnC and myosin binding to thin filaments. Combined with a FLTPR capable of measuring the lifetimes of 1,536 samples in minutes, this makes IANBD-cTnC a useful probe for use in mechanistic studies and HTS enabling therapeutic discovery. This platform allows for the screening of compound libraries to identify (1) drug candidates that modulate cTnC binding to Ca^2+^ and (2) drug candidates that modulate myosin, actin, Tm, cTnT, cTnI, and cTnC—the myofilament proteins responsible for transmitting the structural changes taking place following myosin binding to actin that result in changes in cTnC structure. Finally, our results suggest that compounds like W7, that desensitize Ca^2+^ binding to cTnC, and Mava that regulates the disordered relaxed (DRX) and ordered super-relaxed (SRX) states of myosin, can be identified by this platform. As cardiac myosin-binding protein C (cMyBP-C) is a known modulator of the SRX state, drugs modulating its function might also be detected by this platform.

### Relation to earlier studies

Compared to earlier work (Little et al., 2011; Little et al., 2012), the lifetime changes are qualitatively the same as the fluorescence intensity changes due to Ca^2+^ or myosin binding. The earlier work by Davis and colleagues (Little et al, 2011, 2012) captured transient changes over time, as the cTnC transitioned from one conformation to the next, as in the dynamic physiological contractile conditions of cardiac muscle. Here, we report lifetimes under steady-state conditions that represent cTnC structural states and index of the activation state of the thin filament. The lifetime, rather than intensity, which measures the excited fluorophore decay over a few nanoseconds, precisely reports the cTnC conformation, as an index of the thin filament activation state, under a variety of conditions. Variables that modulate this index include Ca^2+^ concentration, myosin binding, drugs, and likely the position of tropomyosin or mutations in myofilament proteins. Human recombinant troponin was exchanged into skinned myofibrils from porcine hearts, which are of similar size and contain similar myofibrillar content, such as β-cardiac myosin content, as human tissue. Porcine tissue can be obtained inexpensively in large quantities (see Materials and methods) but human tissue studies are also feasible (Dvornikov et al., 2016). Both adult human troponin and mature porcine tissue were used in efforts to increase translational (e.g., physiological conditions, disease mutations, testing of therapeutics) impact in present and future studies. For example, use of adult protein isoforms is an advantage over approaches, such as those centered on the current use of human iPSC-derived cardiomyocytes containing fetal cardiac troponin I (Herron et al., 2016; Wheelwright et al., 2020; Querceto et al., 2022). The method presented here should also be adaptable to *transient* lifetime measurements (Nesmelov et al., 2011), using stopped-flow mixing of myofibrils (Walklate et al., 2022) with physiological variables, including Ca^2+^ and nucleotides, as has been performed with fluorescent probes and actomyosin (Nesmelov et al., 2011; Muretta et al., 2013; Rohde et al., 2018). While most of our comparisons are presented relative to the rigor state for simplicity and mock screening purposes, monitoring the relative changes from ADP or ATP initial states are also feasible.

### Use of myofibrils in HTS

We demonstrate that IANBD-cTnC^T53C^ in the troponin complex can be incorporated into the permeabilized myofibrils by mass action exchange at a level of ∼50% and without loss of function (Fig. 1). This permitted a sufficient signal-to-noise ratio needed for a high quality assay. Permeabilized myofibrils is an accessible model to study the function of ensembles of sarcomeric proteins in cardiac muscle in a plate-reader format. Labeled myofibrils can be loaded into wells in various physiological conditions (Fig. 2). Small compounds can be added to the wells and the plate can be read multiple times. This provides an opportunity to study numerous variables in paired or unpaired formats (Fig. 4). Extensive earlier studies of labeled cTnC^T53C^ exchanged into skinned trabeculae and myofibrils confirmed that these preparations behave biochemically and physiologically similar to wild-type and endogenous cTnC, including measurements of Ca^2+^-dependent increases in force development (Davis et al., 2007; Pinto et al., 2011; Little et al., 2012). The present work demonstrates an excellent high throughput first-pass screen to detect with high precision potential hits of drugs/small-molecules on myofilament regulation that would need to be then validated in more physiologically rigorous settings.

### cTnC fluorescent lifetime probe

Fluorescence intensity of IANBD attached to residue 53 in cTnC^T53C^ has previously been shown to change upon binding to Ca^2+^ and to be responsive to myosin binding to thin filaments when incorporated in myofibrils (Little et al., 2012). Intensity changes result from changes in the environment of IANBD that affect the probe lifetime. In this work, we demonstrate that we can measure lifetime changes of the IANBD probe as cTnC responds directly to Ca^2+^ binding or indirectly to myosin binding to the thin filament. Compared to intensity, in a multi-well plate format, the lifetime measurement is 20-fold more precise and reproducible (Fig. 2, A–D). This improvement with lifetime in comparison to intensity measurements has been shown previously (Cornea et al., 2013), and is due to lifetime being relatively concentration independent and not subject to small variations, such as those arising from pipetting, that affect intensity measurements, which are directly correlated with the amount of fluorophore added to each sample well. Low variation and adequate separation of signals are critical for successful HTS designed to identify compounds that alter structural states of target proteins. Our results indicate that we have achieved the necessary conditions in myofibrils to screen for compounds that modulate the Ca^2+^-free or Ca^2+^-bound states of cTnC. Additionally, the screening for compounds that modulate the propagation of changes in the thin filament responding to myosin binding actin are now feasible in myofibrils. As the IANBD probe was excited by a 473-nm microchip laser, we would expect ∼3% of the small-molecule compounds in a chemical library to be fluorescent themselves and removed as false positives. This estimate is based on work by Schaaf et al. (2018), using the Library of Pharmacologically Active Compounds (LOPAC, from Invitrogen).

### Ca^2+^ and myosin effects on cTnC

To demonstrate the broad application of the cTnC myofibril assay, we successfully performed mock screening assays with variables of low versus high Ca^2+^, ATP versus rigor or ADP, and DMSO versus Mava or W7. Positive *Z*′ factors indicate that all these variables would be reliably detected by cTnC lifetime, suggesting that hit compounds affecting various physiological or pathological states of functioning myofibrils could be readily detected using this assay.

In striated muscle, Ca^2+^ binding to cTnC is accompanied by tropomyosin transitioning from the blocked to the closed state. This exposes actin to myosin binding that results in tropomyosin transitioning further to the open state (McKillop and Geeves, 1993). In the reverse direction, myosin binding to the thin filament increases the affinity of cTnC for Ca^2+^ (Bremel and Weber, 1972; Zot and Potter, 1989). It might then have been predicted that intensity and lifetime changes of a sensor on cTnC would report similarly for both effects, Ca^2+^ and myosin binding. Contrary to this prediction, Ca^2+^ binding (to cTnC) decreased the IANBD intensity, while myosin binding increased the intensity (Davis et al., 2007; Little et al., 2012). We similarly find a decrease in IANBD lifetime upon Ca^2+^ binding and an increase in lifetime with myosin binding (Fig. 2 B). We interpret these results to indicate that when cTnC is bound to Ca^2+^, the IANBD probe attached to residue 53 is in a different environment due to different protein–protein interactions (or structural conformations) than it is in the absence of Ca^2+^ or when the thin filament is impacted by myosin binding. More broadly, this result suggests that the response of cTnC to myosin binding to the thin filament is fundamentally different from its response to binding Ca^2+^.

Caution should be taken to not over-interpret the relative magnitudes or directions of changes in lifetimes of IANBD-cTnC under the various conditions. Different structural/conformational changes in cTnC, arising from many sources, may result in the same direction (increase or decrease) and similar magnitudes of changes in IANBD lifetime. Additionally, changes in conformations of TnC may or may not result in different lifetimes.

We observed nuanced lifetime changes that are sensitive to a range of environmental effectors of acto-myosin contractile function. Intermediate changes in lifetime are detectable at intermediate concentrations of Ca^2+^ where developed force would also be intermediate (i.e., sub-maximal compared to pCa 4.5; Fig. 3). Changes in lifetime upon Ca^2+^ binding was seen in both rigor and nucleotide bound states. In low Ca^2+^, IANBD-TnC not only distinguishes between rigor and the weak-binding state (in the presence of ATP) but also between the proposed SRX and DRX myosin states, the latter being promoted by the drug Mava (Fig. 5 A). These findings of different changes relative to rigor are consistent with earlier results (Bremel and Weber, 1972; Zot and Potter, 1989).

### Ca^2+^ desensitizer W7 and Ca^2+^-sensitizer Pimo

To further validate the cTnC probe and shed light on the effects of Ca^2+^ on IANBD-TnC^T53C^ lifetime, we tested known small-molecule regulatory protein effectors of TnC-Ca^2+^ binding using W7, a Ca^2+^ desensitizer, and Pimo, a Ca^2+^ sensitizer. W7 binds cTnC and directly decreases Ca^2+^ binding affinity (Oleszczuk et al., 2010) and acts on the sarcomere by decreasing the Ca^2+^-sensitivity of force development in skinned cardiomyocytes and reducing the amplitude of contraction in intact cardiomyocytes (Hidaka et al., 1980; Frampton and Orchard, 1992). In contrast, Pimo targets cTnC to increase the Ca^2+^-sensitivity of myofilaments (Sata et al., 1995; Hwang and Sykes, 2015). Here, we observed effects of W7 and Pimo on the IANBD-cTnC lifetime in skinned myofibrils. These opposing effects of W7 and Pimo are detectable at both low and high Ca^2+^ (Fig. 4). W7 would be readily identified in such an HTS with positive *Z*′ at both low and high Ca^2+^ (Fig. 5 B, Tables S1, and S2).

### Myosin inhibitor Mava

We used Mava to test the ability of our assay to detect drugs that modulate interactions between myosin and thin filaments. Our results suggest that the balance between DRX and SRX myosin in low Ca^2+^ has a structural effect on cTnC. In myofilaments, in low Ca^2+^, rigor bound myosin is released from the actin-containing thin filament upon the addition of ATP. This is accompanied by a decrease in IANBD-cTnC^T53C^ lifetime (Fig. 2 B) and intensity (Little et al., 2012). In these conditions, myosin is predicted to be in a mixture of the DRX and SRX states. Mava, a myosin inhibitor that promotes the SRX state (Rohde et al., 2018; Gollapudi et al., 2021), further reduced the IANBD-cTnC^T53C^ lifetime. This suggests that in the ATP, low Ca^2+^ conditions, weak binding myosin interactions due to DRX myosin interacting with the thin filament influence the structural state of cTnC and that cTnC’s structure is changed by promoting SRX myosin.

At high Ca^2+^ with ATP present, Mava again potentiated the myosin detachment effect of ATP and reduced the lifetime of IANBD-cTnC^T53C^. In the absence of Mava, in high Ca^2+^, the 0.1 mM ATP is converted to ADP resulting in myosin strongly bound to the thin filament. With Mava present, the ATP hydrolysis rate is reduced, ATP remains (Rohde et al., 2018; Fig. 1 C), and consequently, more myosin heads are detached from actin. This supports the use of this assay under activating conditions to identify and study compounds modulating the complex interaction of this system. Our new assay has the capacity to identify other drugs that, like Mava, promote the SRX state of myosin.

### Myosin activator OM

We further speculate that the assay will be able to detect conditions and compounds that promote DRX myosin (or the myosin–actin–Tm–Tn interactions that it promotes) and this will be seen as an increase in IANBD-cTnC^T53C^ lifetime in low calcium with ATP present. The myosin activator OM increases myosin’s ATPase activity and stabilizes myosin in the pre-powerstroke conformation (Yotti et al., 2019). OM showed a small (1.1%) but significant (P = 0.004 [average for two experiments]) increase in IANBD-cTnC^T53C^ lifetime at low Ca^2+^ (Table S3), consistent with a distinct mechanism of action that is opposite to Mava.

### In conclusion

Our assay adds to a very small number of cTnC fluorescence-based assays conducive to HTS for therapeutic compounds to treat heart failure (Vetter et al., 2020; Parijat et al., 2021). At this time, it is unclear which screening platform will be most efficacious. Having multiple options of targets to modulate contractile force, like the one we describe here, will undoubtedly increase the chances of success. Our assay satisfies an initial priority to identify compounds that modulate cardiac muscle contractility through interactions with any of numerous myofilament target(s). This assay using myofibrils with reconstituted Tn in a multi-well format is well-suited to studying multiple interventions (e.g., drugs, mutations in the Tn proteins, and phosphorylation status of cTnI) involved in regulating contractility in cardiac muscle.

## Supplementary Material

Table S1 shows IANBD-cTnC^T53C^ fluorescence lifetime changes due to W7 and Pimo in low Ca^2+^

Table S2 shows IANBD-cTnC^T53C^ fluorescence lifetime changes due to W7 and Pimo in high Ca^2+^

Table S3 shows IANBD-cTnC^T53C^ fluorescence lifetime changes due to Mava and OM in low Ca^2+^

Table S4 shows IANBD-cTnC^T53C^ fluorescence lifetime changes due to Mava and OM in high Ca^2+^

Table S5 shows IANBD-cTnC^T53C^ fluorescence lifetime changes due to Mava and OM by well-to-well analysis at high Ca^2+^

SourceData F1 is the source file for Fig. 1.

## Data Availability

All data discussed are presented within the article.
