# Supplementary material for: Fluorescence lifetime-based assay reports structural changes in cardiac muscle mediated by effectors of contractile regulation
Source: J Gen Physiol. 2023 Jan 12;155(3):e202113054. doi: 10.1085/jgp.202113054 (PMC9859762; doi:10.1085/jgp.202113054)
Supplement: Table S1 — shows IANBD-cTnCT53C fluorescence lifetime changes due to W7 and Pimo in low Ca2+ [file JGP_202113054_TableS1.docx]

**Table S1:** IANBD-cTnC^T53C^ fluorescence lifetime changes due to W7 and Pimo in low Ca^2+^

| **Expt.**  **(N)** | **Buffer**  **Condition** | **Buffer**  **Condition** | **Average Lifetime** | **S.D.** | **C.V.** | ***n*** | **Change**  **+Drug** | ***Z′*** | **p=** |
| --- | --- | --- | --- | --- | --- | --- | --- | --- | --- |
| #1 | DMSO | Low Ca^2+^ | 2.36 | 0.02 | 0.6% | 23 |  |  |  |
|  | W7 | Low Ca^2+^ | 2.65 | 0.01 | 0.5% | 24 | 12.3% | 0.71 | 1.5x10^-47^ |
|  | Pimo | Low Ca^2+^ | 2.33 | 0.02 | 0.7% | 24 | -1.1% | -2.7 | 1.7x10^-6^ |
| #2 | DMSO | Low Ca^2+^ | 2.66 | 0.01 | 0.5% | 23 |  |  |  |
|  | W7 | Low Ca^2+^ | 2.96 | 0.01 | 0.3% | 24 | 11.4% | 0.76 | 3.1x10^-51^ |
|  | Pimo | Low Ca^2+^ | 2.57 | 0.01 | 0.3% | 24 | -3.2% | 0.22 | 2.3x10^-28^ |
| Average | DMSO | Low Ca^2+^ | - | - | - |  |  |  |  |
|  | W7 | Low Ca^2+^ | - | - | - |  | 11.8% | 0.73 | 7.4x10^-48^ |
|  | Pimo | Low Ca^2+^ | - | - | - |  | -2.1% | -1.25 | 8.4x10^-7^ |

Average data are provided for individual experiments. Experiments were done with 2 separate protein preparations of troponin that was exchanged into 2 separate myofibril preparations. Low Ca^2+^ is pCa 9. The unit for Average (Fluorescence) Lifetime and S.D. (standard deviation) is nanoseconds (ns). *n* = number of wells of myofibrils into which DMSO, W7, or Pimo is individually added in Rigor buffer at low Ca^2+^. Change +Drug is the % change in lifetime between DMSO and W7 or Pimo for each Experiment. C.V. is the coefficient of variance. Statistical tests of *Z′* factor and t-test are used to evaluate the change in Lifetime between addition of DMSO or DMSO+Drug in low Ca^2+^. The average *Z′* and % Change +Drug for the 2 experiments is also given.
