# Supplementary material for: Fluorescence lifetime-based assay reports structural changes in cardiac muscle mediated by effectors of contractile regulation
Source: J Gen Physiol. 2023 Jan 12;155(3):e202113054. doi: 10.1085/jgp.202113054 (PMC9859762; doi:10.1085/jgp.202113054)
Supplement: Table S2 — shows IANBD-cTnCT53C fluorescence lifetime changes due to W7 and Pimo in high Ca2+ [file JGP_202113054_TableS2.docx]

**Table S2:** IANBD-cTnC^T53C^ fluorescence lifetime changes due to W7 and Pimo in high Ca^2+^

| **Expt.**  **(N)** | **Buffer**  **Condition** | **Buffer**  **Condition** | **Average Lifetime** | **S.D.** | **C.V.** | ***n*** | **Change**  **+Drug** | ***Z′*** | **p=** |
| --- | --- | --- | --- | --- | --- | --- | --- | --- | --- |
| #1 | DMSO | High Ca^2+^ | 2.16 | 0.02 | 0.7% | 23 |  |  |  |
|  | W7 | High Ca^2+^ | 2.28 | 0.01 | 0.6% | 24 | 5.6% | 0.24 | 2.2x10^-29^ |
|  | Pimo | High Ca^2+^ | 2.11 | 0.01 | 0.7% | 24 | -2.4% | -0.75 | 2.8x10^-15^ |
| #2 | DMSO | High Ca^2+^ | 2.42 | 0.01 | 0.6% | 24 |  |  |  |
|  | W7 | High Ca^2+^ | 2.57 | 0.02 | 0.6% | 24 | 6.1% | 0.37 | 1.5x10^-33^ |
|  | Pimo | High Ca^2+^ | 2.36 | 0.01 | 0.6% | 24 | -2.5% | -0.42 | 6.1x10^-19^ |
| Average | DMSO | High Ca^2+^ | - | - | - |  |  |  |  |
|  | W7 | High Ca^2+^ | - | - | - |  | 5.9% | 0.30 | 1.1x10^-29^ |
|  | Pimo | High Ca^2+^ | - | - | - |  | -2.5% | -0.58 | 1.4x10^-15^ |

Average data are provided for individual experiments. Experiments were done with 2 separate protein preparations of troponin that was exchanged into 2 separate myofibril preparations. High Ca^2+^ is pCa 4.5. The unit for Average (Fluorescence) Lifetime and S.D. (standard deviation) is nanoseconds (ns). *n* = number of wells of myofibrils into which DMSO, W7, or Pimo is individually added in Rigor buffer at high Ca^2+^. Change +Drug is the % change in lifetime between DMSO and W7 or Pimo for each Experiment. C.V. is the coefficient of variance. Statistical tests of *Z′* factor and t-test are used to evaluate the change in Lifetime between addition of DMSO or DMSO+Drug in high Ca^2+^. The average *Z′* and % Change +Drug for the 2 experiments is also given.
