# Supplementary material for: Fluorescence lifetime-based assay reports structural changes in cardiac muscle mediated by effectors of contractile regulation
Source: J Gen Physiol. 2023 Jan 12;155(3):e202113054. doi: 10.1085/jgp.202113054 (PMC9859762; doi:10.1085/jgp.202113054)
Supplement: Table S3 — shows IANBD-cTnCT53C fluorescence lifetime changes due to Mava and OM in low Ca2+ [file JGP_202113054_TableS3.docx]

**Table S3:** IANBD-cTnC^T53C^ fluorescence lifetime changes due to Mava and OM in low Ca^2+^

| **Expt.**  **(N)** | **Buffer**  **Condition** | **Buffer**  **Condition** | **Average Lifetime** | **S.D.** | **C.V.** | ***n*** | **Change**  **+Drug** | ***Z′*** | **p=** |
| --- | --- | --- | --- | --- | --- | --- | --- | --- | --- |
| #1 | ATP | Low Ca^2+^ | 2.61 | 0.01 | 0.5% | 23 |  |  |  |
|  | ATP+Mava | Low Ca^2+^ | 2.49 | 0.02 | 0.7% | 23 | -4.4% | 0.19 | 1.6x10^-27^ |
|  | ATP+OM | Low Ca^2+^ | 2.65 | 0.04 | 1.4% | 23 | 1.6% | -2.71 | 1.1x10^-5^ |
| #2 | ATP | Low Ca^2+^ | 2.53 | 0.01 | 0.5% | 23 |  |  |  |
|  | ATP+Mava | Low Ca^2+^ | 2.36 | 0.01 | 0.6% | 23 | -6.5% | 0.51 | 7.0x10^-37^ |
|  | ATP+OM | Low Ca^2+^ | n.d. | n.d. | n.d. | n.d. | n.d. | n.d. | n.d. |
| #3 | ATP | Low Ca^2+^ | 2.41 | 0.01 | 0.4% | 11 |  |  |  |
|  | ATP+Mava | Low Ca^2+^ | 2.19 | 0.03 | 1.3% | 24 | -9.1% | 0.47 | 6.4x10^-23^ |
|  | ATP+OM | Low Ca^2+^ | 2.43 | 0.02 | 0.7% | 24 | 0.63% | -4.30 | 7.7x10^-3^ |
| Average | ATP | Low Ca^2+^ | - | - | - |  |  |  |  |
|  | ATP+Mava | Low Ca^2+^ | - | - | - |  | -6.7% | 0.39 | 2.1x10^-23^ |
|  | ATP+OM | Low Ca^2+^ | - | - | - |  | 1.1% | -3.50 | 3.9x10^-3^ |

Average data are provided for individual experiments. Experiments were done with 2 separate protein preparations of troponin that was exchanged into 3 separate myofibril preparations. Low Ca^2+^ is pCa 9. The unit for Average (Fluorescence) Lifetime and S.D. (standard deviation) is nanoseconds (ns). Variables not determined are denoted with n.d. *n* = number of wells of myofibrils into which ATP, ATP+Mava, or ATP+OM and low Ca^2+^ is individually added in Rigor buffer. Change +Drug is the % change in lifetime between DMSO and Mava or OM for each Experiment. C.V. is the coefficient of variance. Statistical tests of *Z′* factor and t-test are used to evaluate the change in Lifetime between addition of ATP or ATP+Drug in low Ca^2+^. The average *Z′* and % Change +Drug for the 3 experiments is also given.
