# Supplementary material for: Fluorescence lifetime-based assay reports structural changes in cardiac muscle mediated by effectors of contractile regulation
Source: J Gen Physiol. 2023 Jan 12;155(3):e202113054. doi: 10.1085/jgp.202113054 (PMC9859762; doi:10.1085/jgp.202113054)
Supplement: Table S4 — shows IANBD-cTnCT53C fluorescence lifetime changes due to Mava and OM in high Ca2+ [file JGP_202113054_TableS4.docx]

**Table S4:** IANBD-cTnC^T53C^ fluorescence lifetime changes due to Mava and OM in high Ca^2+^

| **Expt.**  **(N)** | **Buffer**  **Condition** | **Buffer**  **Condition** | **Average Lifetime** | **S.D.** | **C.V.** | ***n*** | **Change**  **+Drug** | ***Z′*** | **p=** |
| --- | --- | --- | --- | --- | --- | --- | --- | --- | --- |
| #1 | ^@^ADP | High Ca^2+^ | 2.42 | 0.02 | 0.8% | 22 |  |  |  |
|  | ATP+Mava | High Ca^2+^ | 2.30 | 0.02 | 0.8% | 23 | -4.9% | 0.04 | 3.3x10^-24^ |
|  | ^@^ADP+OM | High Ca^2+^ | 2.44 | 0.03 | 1.1% | 23 | 0.9% | -5.26 | 2.9x10^-3^ |
| #2 | ^@^ADP | High Ca^2+^ | 2.36 | 0.02 | 0.9% | 23 |  |  |  |
|  | ATP+Mava | High Ca^2+^ | 2.26 | 0.02 | 1.0% | 23 | -4.0% | -0.35 | 5.4x10^-19^ |
|  | ^@^ADP+OM | High Ca^2+^ | n.d. | n.d. | n.d. | n.d. | n.d. | n.d. | n.d. |
| #3 | ^@^ADP | High Ca^2+^ | 2.28 | 0.03 | 1.4% | 24 |  |  |  |
|  | ATP+Mava | High Ca^2+^ | 2.19 | 0.04 | 1.6% | 24 | -4.1% | -1.19 | 2.2x10^-12^ |
|  | ^@^ADP+OM | High Ca^2+^ | n.d. | n.d. | n.d. | n.d. | n.d. | n.d. | n.d. |
| #4 | ^@^ADP | High Ca^2+^ | 2.36 | 0.03 | 1.4% | 12 |  |  |  |
|  | ATP+Mava | High Ca^2+^ | 2.26 | 0.02 | 0.7% | 12 | -4.2% | -0.41 | 1.8x10^-9^ |
|  | ^@^ADP+OM | High Ca^2+^ | 2.40 | 0.03 | 1.3% | 12 | 2% | -2.99 | 1.3x10^-3^ |
| Average | ^@^ADP | High Ca^2+^ | - | - | - |  |  |  |  |
|  | ATP+Mava | High Ca^2+^ | - | - | - |  | -4.3% | -0.48 | 4.4x10^-10^ |
|  | ^@^ADP+OM | High Ca^2+^ | - | - | - |  | 1.5% | -4.13 | 2.1x10^-3^ |

Average data are provided for individual experiments. Experiments were done with 3 separate protein preparations of troponin that was exchanged into 4 separate myofibril preparations. High Ca^2+^ is pCa 4.5. The unit for Average (Fluorescence) Lifetime and S.D. (standard deviation) is nanoseconds (ns). Variables not determined are denoted with n.d. *n* = number of wells of myofibrils into which ATP, ATP+Mava, or ATP+OM and high Ca^2+^ is individually added in Rigor buffer. Change +Drug is the % change in lifetime between DMSO and Mava or OM for each Experiment. C.V. is the coefficient of variance. Statistical tests of *Z′* factor and t-test are used to evaluate the change in Lifetime between addition of ATP or ATP+Drug in high Ca^2+^. ^@^At 20 min, ATP was hydrolyzed to ADP in control and OM samples. The average *Z′* and % Change +Drug for the 4 experiments is also given.
