# Supplementary material for: Fluorescence lifetime-based assay reports structural changes in cardiac muscle mediated by effectors of contractile regulation
Source: J Gen Physiol. 2023 Jan 12;155(3):e202113054. doi: 10.1085/jgp.202113054 (PMC9859762; doi:10.1085/jgp.202113054)
Supplement: Table S5 — shows IANBD-cTnCT53C fluorescence lifetime changes due to Mava and OM by well-to-well analysis at high Ca2+ [file JGP_202113054_TableS5.docx]

**Table S5:** IANBD-cTnC^T53C^ fluorescence lifetime changes due to Mava and OM by well-to-well analysis at high Ca^2+^

| **Expt.**  **(N)** | **Buffer**  **Condition** | **Buffer**  **Condition** | **Avg. Change from Rigor** | **S.D.** | ***n*** | **Change**  **+ Drug** | ***Z′*** | **p=** |
| --- | --- | --- | --- | --- | --- | --- | --- | --- |
| #1 | ^@^ADP | High Ca^2+^ | -0.2% | 1% | 22 |  |  |  |
|  | ATP+Mava | High Ca^2+^ | -6.4% | 0.7% | 24 | -6.2% | 0.27 | 8.5x10^-30^ |
|  | ^@^ADP+OM | High Ca^2+^ | -0.5% | 1% | 23 | 0.7% | -7.47 | 0.0024 |
| #2 | ^@^ADP | High Ca^2+^ | -2.4% | 0.7% | 23 |  |  |  |
|  | ATP+Mava | High Ca^2+^ | -6.8% | 0.7% | 23 | -4.4% | -0.07 | 8.8x10^-23^ |
|  | ^@^ADP+OM | High Ca^2+^ | n.d. | n.d. | n.d. | n.d. | n.d. | n.d. |
| #3 | ^@^ADP | High Ca^2+^ | -4.6% | 1.0% | 24 |  |  |  |
|  | ATP+Mava | High Ca^2+^ | -11.3% | 0.7% | 24 | -6.7% | 0.25 | 1.5x10^-29^ |
|  | ^@^ADP+OM | High Ca^2+^ | n.d. | n.d. | n.d. | n.d. | n.d. | n.d. |
| #4 | ^@^ADP | High Ca^2+^ | -0.5% | 1.0% | 11 |  |  |  |
|  | ATP+Mava | High Ca^2+^ | -7.0% | 0.3% | 12 | -6.5% | 0.39 | 3.0x10^-16^ |
|  | ^@^ADP+OM | High Ca^2+^ | -0.1% | 1.0% | 12 | 0.5% | -10.4 | 0.021 |
| Average | ^@^ADP | High Ca^2+^ | - | - |  |  |  |  |
|  | ATP+Mava | High Ca^2+^ | - | - |  | -5.9% | 0.21 | 7.5x10^-17^ |
|  | ^@^ADP+OM | High Ca^2+^ | - | - |  | 0.6% | -8.92 | 0.012 |

Average data are provided for individual experiments. Experiments were done with 3 separate protein preparations of troponin that was exchanged into 4 separate myofibril preparations. High Ca^2+^ is pCa 4.5. Variables not determined are denoted with n.d. Well-to-well analysis is performed by first measuring cTnC fluorescence lifetime in myofibrils in rigor buffer with Ca^2+^ and drug (DMSO control, Mava, or OM), and then measuring the percent change in each well following addition of ATP, in the re-scan for fluorescence lifetime. S.D. is standard deviation. Variables not determined are denoted with n.d. *n* = number of wells of myofibrils into which ATP, ATP+Mava, or ATP+OM and high Ca^2+^ is individually added in Rigor buffer. Change +Drug is the % change in lifetime between DMSO and Mava or OM for each Experiment. Statistical tests of *Z′* factor and t-test are used to evaluate the change in Lifetime between addition of ATP or ATP+Drug in high Ca^2+^. ^@^At 20 min, ATP was hydrolyzed to ADP in control and OM samples. The average *Z′* and % Change +Drug for the 4 experiments is also given.
