# Supplementary figures and images for: Fluorescence lifetime-based assay reports structural changes in cardiac muscle mediated by effectors of contractile regulation
Source: J Gen Physiol. 2023 Jan 12;155(3):e202113054. doi: 10.1085/jgp.202113054 (PMC9859762; doi:10.1085/jgp.202113054)

**B** Coomassie stain (Main text):

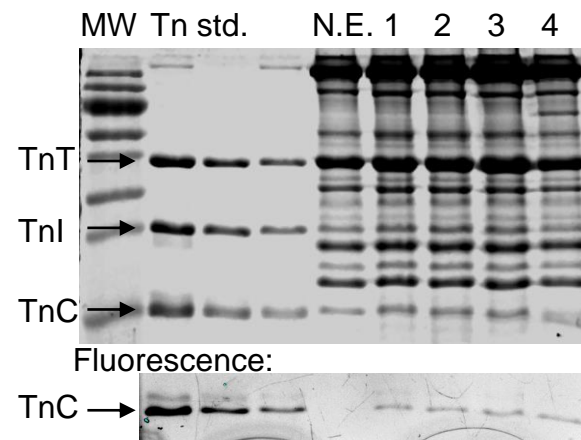

**B** Coomassie stain ([Source Data](#)):

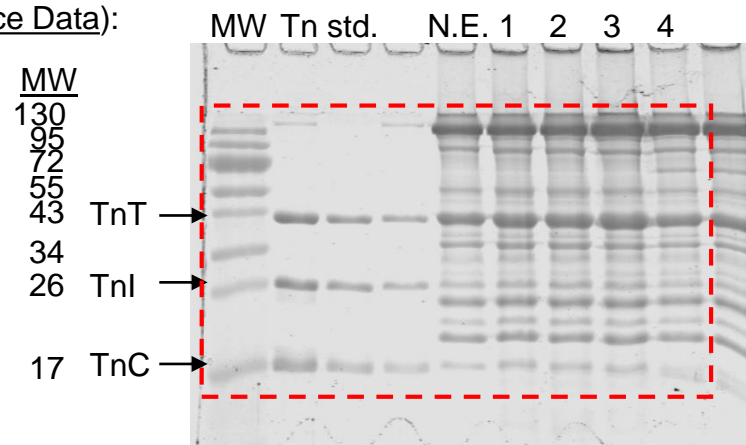

Fluorescence ([Source Data](#)):

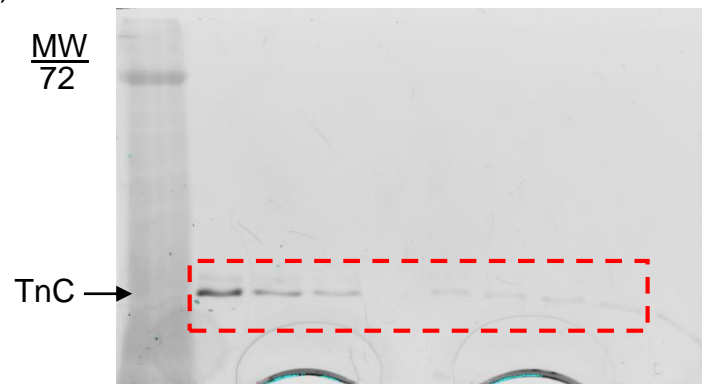

Supplement: SourceData F1 — is the source file for Fig. 1. [file JGP_202113054_SourceDataF1.pdf]
